# A new family of TonB-dependent copper transporters linked to respiratory oxidase function

**DOI:** 10.1016/j.jbc.2026.111180

**Published:** 2026-01-20

**Authors:** Majda Hachmi, Manon Mirgaux, René Wintjens, Chloé Carassus, Pascal Arnoux, Gauthier Roy, Alex Rivera-Millot, Stéphanie Slupek, Anne-Sophie Debrie, Véronique Alaimo, Gabriel Billon, Loïc Coutte, Rudy Antoine, Françoise Jacob-Dubuisson

**Affiliations:** 1 University of Lille, Inserm, CNRS, CHU Lille, Institut Pasteur de Lille, U1019-UMR9017-CIIL-Center for Infection and Immunity of Lille, Lille, France; 2 Université Libre de Bruxelles, Faculty of Pharmacy, Department of Research in Drug Development, Unit of Microbiology, Bioorganic and Macromolecular Chemistry, Bruxelles, Belgium; 3 Aix Marseille University, CEA, CNRS, BIAM, Saint-Paul-lez-Durance, France; 4 CNRS, UMR8516 Laboratoire de Spectroscopie pour les Interactions, la Réactivité et l’Environnement, Université de Lille, Lille, France

**Keywords:** *Bordetella pertussis*, copper transport, crystal structure, membrane protein, outer membrane, respiratory oxidases, sequence similarity network, TonB-dependent transporter

## Abstract

Copper is an essential metal notably found in respiration complexes for its redox properties. It is also toxic hence its cellular trafficking is tightly controlled. Bacteria have developed a number of defense systems against copper excess, but its acquisition pathways remain poorly characterized. Ubiquitous in Gram-negative bacteria, TonB-dependent transporters (TBDTs) are outer membrane β-barrel proteins that mediate the proton motive force-dependent import of various nutrients to the periplasm. Here, we characterized a TBDT that imports copper in the whooping cough agent *Bordetella pertussis*, CrtA^Bp^ (formerly BfrG), which is a prototype of a new subfamily of TBDTs. Our data indicate that CrtA^Bp^ is dedicated to the import of copper for heme-copper respiratory oxidoreductases. We revealed that CrtA^Bp^ imports chelated rather than free copper, solved the crystal structure of CrtA^Bp^ and identified a conserved ligand binding site. By combining bacterial growth experiments, biophysical approaches and AlphaFold3 modeling we sketched out the features of copper-ligand complexes for CrtA^Bp^. In contrast with ferrisiderophore-specific TBDTs, no high-affinity chalkophore ligand of CrtA^Bp^ could be identified, implying two nonmutually exclusive models. In the host, CrtA^Bp^ might use a xenometallophore produced by another species present in the same niche to acquire copper. *In vitro* however, CrtA appears not to have high-affinity ligands but to import copper chelated by small molecules notably harboring carboxylate groups, which might represent a paradigm of ‘scavenger’ TBDTs with low ligand selectivity. We identified an essential, invariant histidine residue that might serve as a selectivity filter for copper-chelate complexes.

Copper (Cu) is used by organisms living in oxygen-rich environments as an important cofactor for redox centers and electron transfer reactions in various processes, including energy generation. Heme-copper oxidoreductase (HCO) cytochrome c oxidases are notably the most frequent cuproproteins in aerobic bacteria (1). Bacteria also employ Cu in superoxide dismutases to neutralize reactive oxygen species produced by phagocytic cells (2). However, the use of copper is associated with dangers. Cu (I) in excess can inactivate essential iron-sulfur cluster enzymes, generate reactive oxygen species and induce protein misfolding and aggregation (3, 4). There is hardly any free copper available in cells, and its intracellular trafficking relies on metal-mediated protein-protein interactions (5).

Bacteria strictly regulate their intracellular copper homeostasis. Various defense systems protect Gram-negative bacteria against its toxicity including extrusion, periplasmic oxidation of Cu(I) into Cu(II) and sequestration in the periplasm or the cytoplasm (3). In the context of infection, host nutritional immunity encompasses both metal intoxication of microorganisms, well documented for Cu, and their starvation of metals such as Fe, Mn and Zn, a concept that has recently emerged for Cu as well (6, 7, 8). However, little is known about copper acquisition in prokaryotes. Cu influx through the cytoplasmic membrane is notably mediated by small mechanosensitive channels (9) or specific transporters (10, 11). Across the outer membrane, passive diffusion *via* porins is considered the primary Cu(II) import pathway in diderm bacteria although this is likely insufficient under copper-limiting conditions (12, 13). Few Cu-specific outer membrane transporters have been identified. They belong to the TonB-dependent transporters (TBDT) superfamily, characterized by a conserved scaffold *i.e.*, a C-terminal 22-stranded β-barrel domain and an N-terminal plug domain that obstructs the barrel. TBDTs mediate the transport of various nutrients using the proton motive force delivered by the inner membrane TonB-ExbBD complex (14). Among copper-importing TBDTs, OprC of *Pseudomonas aeruginosa* and MbnT of *Methylosinus trichosporium* import naked copper ions and copper ions in complex with modified peptides called methanobactins, respectively (15, 16). Copper can also be imported in complex with the broad-spectrum siderophore yersiniabactin through the YbtA/FyuA TBDT (17).

*Bordetella pertussis* is a strictly aerobic, Gram-negative bacterium responsible for whooping cough. It produces virulence factors controlled by the two-component system BvgAS to colonize the human upper respiratory epithelium (18, 19). With respect to copper homeostasis *B. pertussis* has streamlined its mechanisms of protection compared with environmental bacteria, most likely because its exposure is low in its restricted niche (20, 21). Transcriptomic analyses have revealed a strong repression of the three-gene operon *cruR-bfrG-bp2921* by copper excess. This regulation is post-transcriptionally mediated by the first gene of the operon coding for the upstream open reading frame CruR (22). CruR homologs are encoded in copper-related operons in various species (22). The second gene encodes BfrG (renamed CrtA^Bp^ in this study), one of the 15 TBDTs of *B. pertussis* and the only one to respond to copper (22). Unlike the genes of FauA, BhuR and BfeA, whose products transport Fe(III) in complex with alcaligin, heme and enterobactin, respectively, the *cruR-crtA-bp2921* promoter lacks a Fur-binding site (23). The third gene of the operon, *bp2921*, codes for a cytoplasmic membrane protein with two large periplasmic domains, which belongs to the PepSY_TM family (Pfam PF03413). The function of the *bp2921* gene product in *B. pertussis* is unknown, but its distant *P. aeruginosa* FoxB homolog was shown to be a reductase of Fe(III) in complex with the siderophore ferrioxamine (24). Here we discovered that BfrG/CrtA^Bp^ is a TBDT that plays a role in copper acquisition most likely for respiratory HCOs.

## Results

### BfrG/CrtA^Bp^ is the prototype of a family of TonB-Dependent Transporters

**Figure 1:**
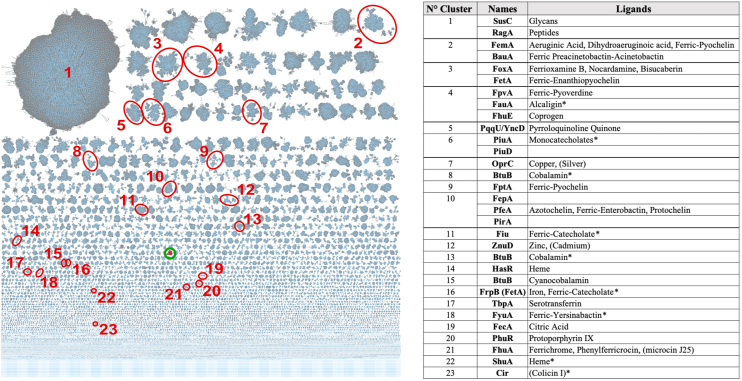
**Sequence similarity analysis of the TBDT superfamily.** The representative node network (AST value = 180) shows the full diversity of the superfamily. To make the analysis possible sequence redundancy was reduced to < 70% (approx. 300,000 proteins left out of > 2 10^6^). The sequence cluster that contains CrtA^Bp^ and its orthologs is circled in *green*. The numbered clusters (circled in *red*) are those for which X-ray structures are available (see Table S1 for the pdb ID numbers). The list at the *right* provides all unique crystallized proteins found in the indicated sequence clusters and their co-crystallized ligands. The *asterisks* indicate examples of known ligands that were not cocrystallized with their respective TBDTs. The parentheses indicate nonnatural or parasitic ligands. The BtuB proteins of Bacteroidota (clusters eight and 13) and Pseudomonadota (cluster 15) form separate clusters according to this analysis. See Fig. S1 for the *B. pertussis* and *P. aeruginosa* TBDTs and for the known copper-importing TBDTs, and Table S2 for for the Genbank ID numbers of all TBDTs found in the labeled clusters.

To determine the position of BfrG/CrtA in the TBDT family tree, we built a sequence similarity network. The representative node network showed hundreds of distinct sequence clusters, a small one of which includes BfrG/CrtA (circled in green in Fig. 1). This sequence cluster is distinct from all the clusters of TBDTs of known structure, from those of known *B. pertussis* TBDTs, and from those characterized in *P. aeruginosa*, including OprC (Figs. 1, S1 and Tables S1 and S2). However, PA0434 of *P. aeruginosa* (OptJ/CrtA^Pa^), which is produced in response to low copper availability (25, 26) belongs to the same sequence cluster as BfrG/CrtA (Fig. S1). As the expression of these proteins is controlled by copper, we propose to name them ‘CrtA’ (copper-responsive transporter A), hence that of *B. pertussis* will be hereafter called CrtA^Bp^. On the premise that transporters of the same cluster are isofunctional, CrtA-type TBDTs likely have a distinct specificity.

**Figure 2:**
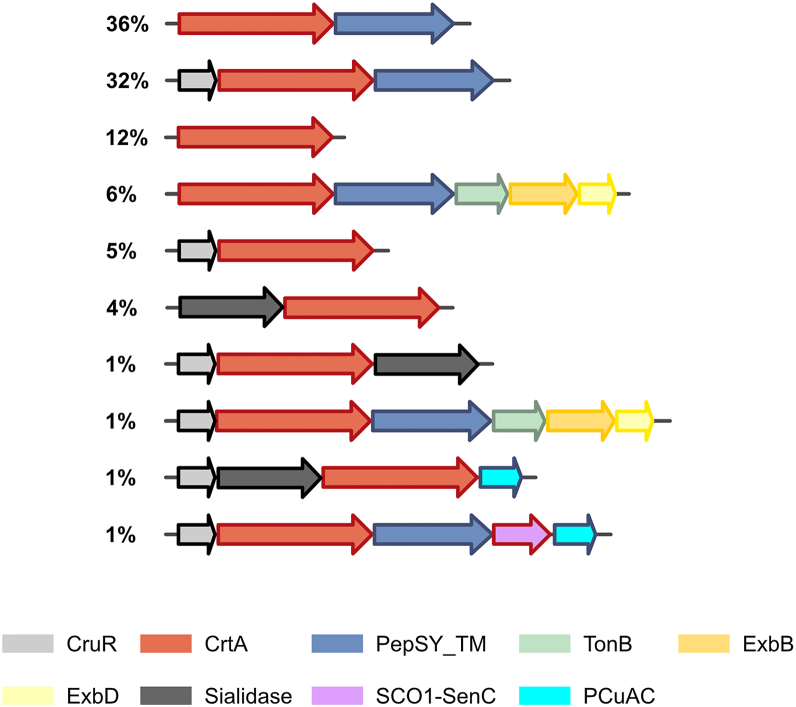
**Genetic organizations of the operons harboring *crtA* orthologs.** The CrtA-coding genes are shown in *red*, the genes most frequently found in genetic association with them are represented in other colors, and the types of proteins encoded are provided below. The frequency at which each genetic organization is found is indicated as a percentage (relative to the number of genomes included in this analysis). See Table S3 for genomic analyses of the bacterial strains that harbor *crtA* genes.

CrtA orthologs are 707 to 911 residues long and mostly found in β- and γ-Proteobacteria. We built a sequence logo which showed no conserved Cys and Met residues and only two conserved His residues, known to preferentially bind Cu(I) and Cu(II), respectively (Fig. S2). We determined the genetic environments of *crtA* orthologs using a Rodeo-type approach (21). In > 75% of the genetic loci, *crtA* genes are followed by genes coding for PepSY_TM-type proteins homologous to the *bp2921* gene product in *B. pertussis* (Fig. 2 and Table S3). More than 40% of the operons harbor *cruR* orthologs in first position, indicating that copper regulation of these operons is widespread (22). ExbBD- and TonB-coding genes were found in some cases, indicating that the corresponding TBDTs may use specific TonB complexes. Genes for the copper chaperones PCuAC and ScoI involved in HCO assembly (27, 28) were found in a few operons (Fig. 2 and Table S3).

### Search for a role of CrtA^Bp^

**Figure 3:**
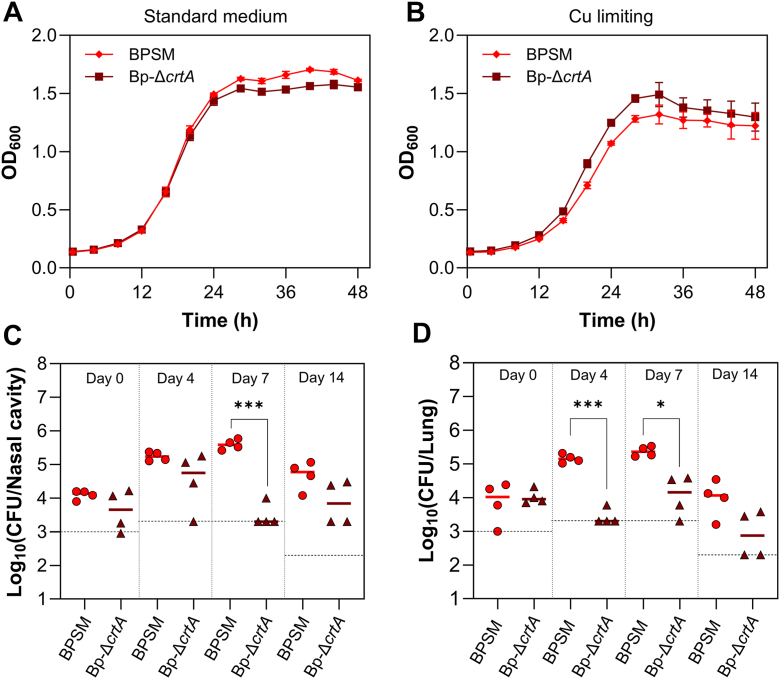
**Role of CrtA *in vitro and in vivo*.***A* and *B,* BPSM and *Bp-ΔcrtA* were grown in SS medium (*A*) or under Cu limitation caused by the addition of 50 μM BCS (*B*). The use of BCS, a Cu(I) chelator, was justified by the reducing power of the SS medium (which contains 2 mM ascorbic acid), making available copper in the Cu(I) form. All growth curves are representative of three biological replicates, with the means and standard deviations (SD) calculated on four technical replicates. *C* and *D,* following coinfection of mice with WT *B. pertussis* and Bp-*ΔcrtA*, bacterial loads were quantified in the nasal cavities (*C*) and lungs (*D*). The two strains were mixed and co-inoculated in equal numbers, using a BPSM variant harboring a gentamycin resistance gene for colony-forming units counts on plates. Each point represents an individual mouse, and the dashed lines indicate the limits of detection. Statistical significance was determined using a non-parametric permutation-based ANOVA (∗, *p* < 0.05; ∗∗∗, *p* < 0.001).

To investigate the potential involvement of CrtA^Bp^ in copper import, we used a mutant strain harboring an in-frame deletion of *crtA*, *Bp-*Δ*crtA*^*Bp*^ (22). We checked the absence of a polar effect of the deletion on the following *bp2921* gene by RT-qPCR (Fig. S3), and we also verified the absence of extraneous mutations by whole genome sequencing. We then compared the growth of *Bp-*Δ*crtA*^*Bp*^ with that of the wt strain called BPSM, in standard medium or with a specific Cu(I) chelator, bathocuproine disulfonate (BCS), added to the cultures to cause copper limitation (21). No growth difference was observed between the mutant strain and its parent (Fig. 3, *A* and *B*). To assess the possibility that *crtA* is in decay in the *B. pertussis* genome, we inspected this gene in all sequenced isolates. It was identical to that of BPSM in 98% cases, appeared to be inactivated by a frameshift mutation in three cases only and was absent from a single strain (Table S4). This strong conservation supports the idea that CrtA^Bp^ plays a role in *B. pertussis*.

We thus tested if CrtA^Bp^ might be required *in vivo* by performing a colonization experiment. Mice were inoculated intranasally with BPSM or *Bp-*Δ*crtA*^*Bp*^. Over the course of the infection, however, similar numbers of WT and mutant bacteria were found in the mice nasal cavities and lungs (Fig. S4). To test the competitiveness of the *Bp-*Δ*crtA*^*Bp*^ strain, we co-infected mice with the mutant strain and its isogenic parent in equal numbers. Under these conditions, *Bp-*Δ*crtA*^*Bp*^ was cleared faster than BPSM from both the mice nasal cavities and lungs (Fig. 3, *C* and *D*). Thus, although CrtA is not essential, it appears to contribute to bacterial fitness *in vivo*.

### Link with respiration

The *crtA*^*Bp*^ gene is close to the HCO operon *cyoABCD (bp2930–33)*. As HCOs are major cuproproteins in *B. pertussis* (20), we asked if CrtA^Bp^’s role might be related to HCO function. *In silico* analyses of *crtA*-harboring genomes showed that they all also harbor HCO-coding genes, and most also have PCuAC- and ScoI-coding genes, indicating that these bacteria assemble HCO complexes (Table S3).

**Figure 4:**
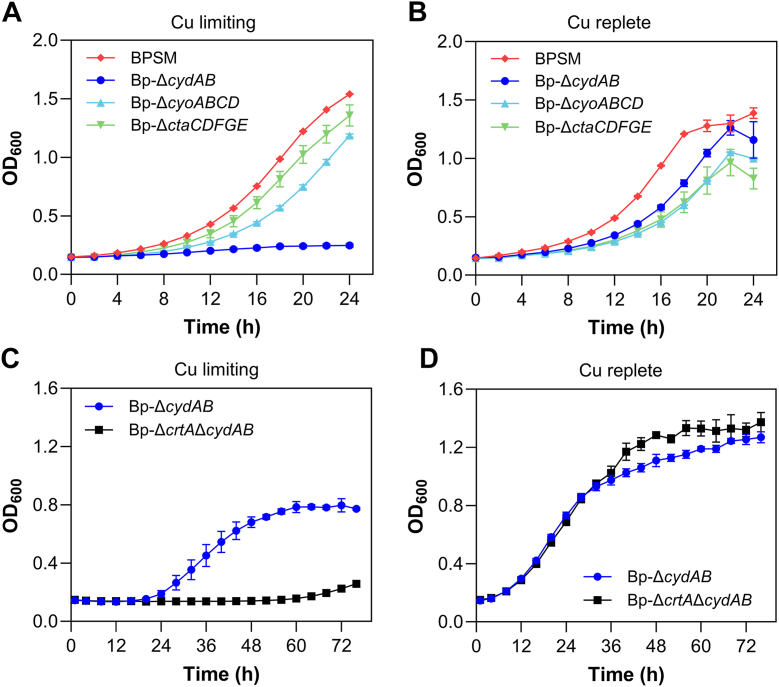
**Growth phenotypes of respiratory mutants.***A* and *B,* growth of BPSM, *Bp-*Δ*cydAB*, *Bp-*Δ*cyoABCD* and *Bp-ΔctaCDEFG* in SS medium supplemented with either 50 μM BCS (copper-limiting condition) (*A*) or 50 μM CuSO_4_ (copper-replete condition) (*B*). Unlike the two copper-dependent HCO mutants, *Bp*-Δ*cydAB* did not grow under copper-limiting conditions, indicating that CydAB function is essential when copper is unavailable. *C* and *D,* the copper-independent cytochrome bd ubiquinol oxidase deletion mutant *Bp-*Δ*cydAB* and the double mutant *Bp-*Δ*crtA*Δ*cydAB* were grown with 15 μM BCS (*C*) or 15 μM BCS + 2 μM CuSO_4_ (*D*). In (*C*), ascorbate present in the SS medium (2 mM) reduces Cu(II) to Cu(I), which is insoluble and chelated by BCS. The long lag phase for *Bp-*Δ*cydAB* likely corresponds to the time necessary for Cu(I) to be gradually re-oxidized in aerated cultures, making Cu(II) available for growth (see Fig. S6 for the effect of ascorbate and BCS on growth). Representative curves of three biological replicates are shown, with the means and SD calculated on three or four technical replicates.

*B. pertussis* possesses two HCOs, the cytochrome bo oxidase CyoABCD and the cytochrome aa3 oxidase CtaCDFGE, as well as a copper-independent cytochrome bd ubiquinol oxidase, CydAB (29). We separately deleted the corresponding three operons and tested the growth of the mutant strains in copper-limiting or copper-replete conditions. The absence of *cydAB* severely impacted growth in copper limitation, unlike that of either of the other two operons, and growth was rescued by copper supplementation (Fig. 4, *A* and *B*). CydAB is thus essential for growth in copper-limiting conditions, whereas in its absence the HCOs sustain growth provided that copper is present.

Taking advantage of the dependence of *Bp-*Δ*cydAB* on copper, we deleted *cydAB* in *Bp-ΔcrtA* and monitored the growth of the resulting strain in copper-limiting (+BCS) or copper-replete (+CuSO_4_) conditions. After 24 h *Bp*-Δ*cydAB* started growing in the presence of BCS, unlike *Bp*-Δ*crtA*Δ*cydAB*, whereas the addition of CuSO_4_ supported the growth of both strains (Fig. 4, *C* and *D*). The growth phenotype of *Bp*-Δ*crtA*Δ*cydAB* was complemented by the expression of *crtA*^Bp^ from a plasmid, although partially (Fig. S5). The long lag in the growth curve of *Bp*-Δ*cydAB* under copper-limiting conditions was reduced if ascorbate was removed from the medium (Fig. S6). We interpret these results as follows. At the start of the culture ascorbate reduces all available Cu(II), and Cu(I) is chelated by BCS. This starves bacteria of copper and hampers growth. Progressive oxidation of ascorbate over time in the strongly aerated cultures makes Cu(II) gradually available, allowing bacterial growth to resume. In copper-replete conditions (Fig. 4*D*) *Bp*-Δ*crtA*Δ*cydAB* and *Bp*-Δ*cydAB* grew similarly, most likely thanks to nonspecific import of Cu(II). Collectively, these results indicated that CrtA^Bp^ imports Cu(II) and appears to be dedicated to HCOs in *B. pertussis*.

### Structure of CrtA^Bp^ and role of the conserved binding site

**Figure 5:**
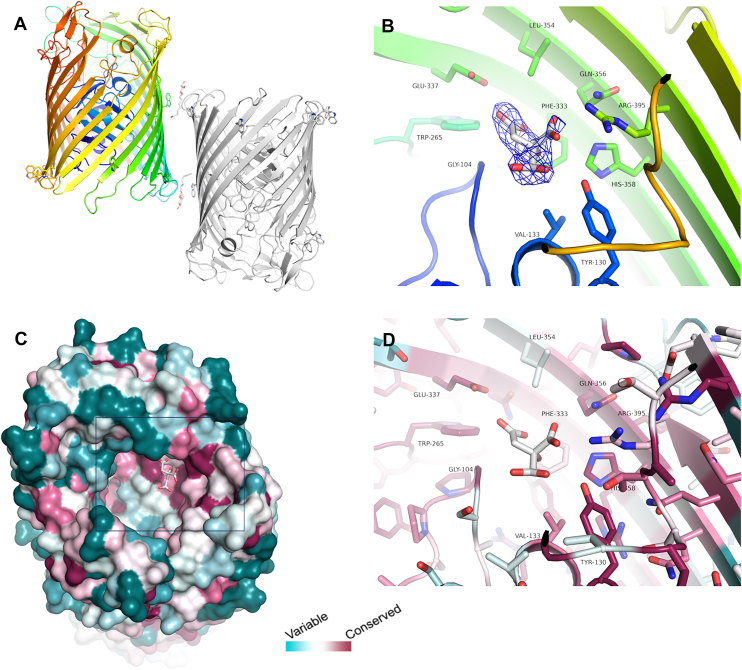
**X-ray structure of CrtA^Bp^.***A,* crystal structure of the two molecules present in the asymmetric unit of the *P*2_1_ space group. One molecule is colored from *blue* at the N-terminus to *red* at the C-terminus while the other is shown in *gray*. Two βOG molecules used during protein purification are visible and help to delineate the membrane-spanning region, along with Trp residues shown as sticks. *B,* structure of CrtA^Bp^ from crystals obtained in the presence of citrate (P1 space group), showing a citrate molecule stabilized on the extracellular side. The electron density around citrate is depicted as of an F_o_-F_c_ omit map contoured at 2σ. Side chains of residues within 4 Å of the citrate are shown as sticks. *C,* surface representation of CrtA^Bp^ as viewed from the extracellular side (*i.e.*, from the top if we refer to the position of the colored monomer in *panel A*), colored according to sequence conservation as defined in consurf. The citrate binding site appears to be relatively well conserved. The *square* indicates the region magnified in *panel* (*D*) showing a close-up view of the citrate binding site with carbon atoms colored by sequence conservation score. Only residues within 4 Å of the citrate are labeled, several of which are conserved.

We determined the structure of recombinant CrtA^Bp^ by X-ray crystallography in two crystal forms (Table S5). CrtA^Bp^ adopts a typical TBDT fold, composed of a 22-strand β barrel comprising residues 178 to 732, obstructed by an N-terminal plug domain encompassing residues 42 to 171 and containing a typical 4-stranded mixed β sheet. The long extracellular β5-β6 and β9-β10 loops fold over the top of the short β20 to β6 barrel strands (Fig. 5*A*). Together with the β13-β14 and β21-β22 loops, they somewhat restrict access to the channel. The structure of the *P*2_1_ crystal form of CrtA^Bp^ solved to 2.9 Å resolution shows the stabilization of two β-octylglucoside (βOG) molecules used to solubilize the protein, which delineate the membrane-spanning region (Fig. 5*A*). A *P*1 crystal form obtained using a citrate-containing buffer and solved to 2.3 Å resolution shows the stabilization of a citrate molecule in a conserved cavity on the extracellular side of CrtA^Bp^ (Fig. 5, *B*–*D*). Although the presence of citrate in CrtA^Bp^ might be a crystallization artefact, its position globally corresponds to those of Fe(III)-siderophore complexes in their respective receptors (30, 31). Furthermore, it was located in the vicinity of residues highly conserved in the CrtA family, namely E337, Q356, H358, R395, W265 and Y130 (Fig. S2). Y130 is part of the plug, W265 belongs to the β5-β6 loop, E337 is in β7, H358 and Q356 in β8 and R395 in β9. Their conservation suggests that they might form a ligand binding site (Fig. 5, *C* and *D*). Attempts to soak the crystals with copper or to co-crystallize CrtA with a Cu-citrate complex were unsuccessful.

**Figure 6:**
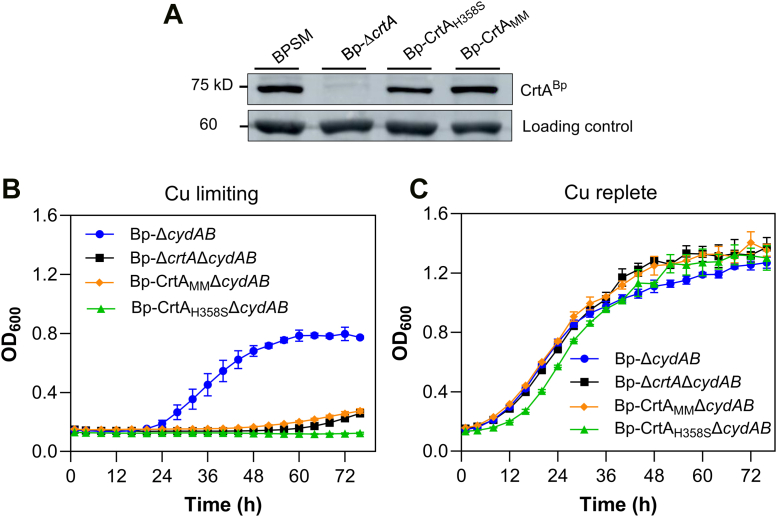
**Importance of the ligand binding site for Cu uptake.***A,* expression of CrtA_MM_ and CtrA_H358S_ from the natural locus in *B. pertussis*. Clarified cell lysates of BPSM, *Bp*-Δ*crtA*, *Bp*-CrtA_H358S_ and *Bp*-CrtA_MM_ were analyzed by immunoblotting using anti-CrtA^Bp^ antibodies. The loading control is a nonspecific protein band from the same immunoblot. *B* and *C, Bp*-Δ*cydAB*, *Bp*-Δ*crtAΔcydAB*, *Bp*-CrtA_MM_*ΔcydAB* and *Bp*-CrtA_H358S_*ΔcydAB* were grown with 15 μM BCS (*B*) or 15 μM BCS + 2 μM CuSO_4_ (*C*). Representative curves of three biological replicates are shown, with the means and SD calculated on three technical replicates.

To probe the importance of this putative binding site, we replaced all six conserved residues with Ala in *B. pertussis*, yielding Bp-CrtA_MM_ (for Multiple Mutant). The substitutions did not affect the production of the transporter as shown by Western blot analysis (Fig. 6*A*). Comparison of the growth phenotype of *Bp-C*rtA_MM_Δ*cydAB* with those of *Bp*-Δ*cydAB* and *Bp-ΔcrtAΔcydAB* under copper-limiting conditions showed that *Bp-C*rtA_MM_Δ*cydAB* phenocopied the deletion mutant (Fig. 6, *B* and *C*). To verify that the substitutions did not affect the folding and/or the stability of the protein, we produced recombinant WT and mutant CrtA^Bp^ and performed thermal unfolding analyses. Similar melting temperatures (Tm) of 71 °C were measured for both proteins, ruling out structural disruptions caused by the substitutions.

Because His residues are prone to coordinate Cu(II) ions, we also introduced a single His358 to Ser substitution and assessed the growth phenotype of the resulting strain, *Bp*-CrtA_H358S_Δ*cydAB.* The single His358-Ser substitution was sufficient to phenocopy *Bp*-Δ*crtA*Δ*cydAB,* demonstrating the pivotal role of the invariant His residue for CrtA^Bp^ function (Fig. 6, *B* and *C*).

### Search for CrtA^^Bp^^ ligands

The serendipitous presence of citrate in the CrtA^Bp^ cavity suggested that the transporter imports Cu(II) in a chelated form. Iron-specific TBDTs use endogenous siderophores or xeno-siderophores available in the same niche as Fe(III) ligands (32) or acquire Fe(III) from host-derived proteins (33). As for CrtA^Bp^, the growth phenotypes shown above suggested that in laboratory conditions it imports Cu(II) in complex with a molecule present in the medium. *B. pertussis* synthesizes two metallophores, the siderophore alcaligin (34) and the chalkophore bufferin (35). However, inactivation of either operon in the *Bp-ΔcydAB* background did not phenocopy *Bp-ΔcrtAΔcydAB* (Fig. S7). *In silico* searches of *crtA*-harboring genomes failed to identify shared biosynthetic gene clusters encoding other known metallophores (Table S3).

**Figure 7:**
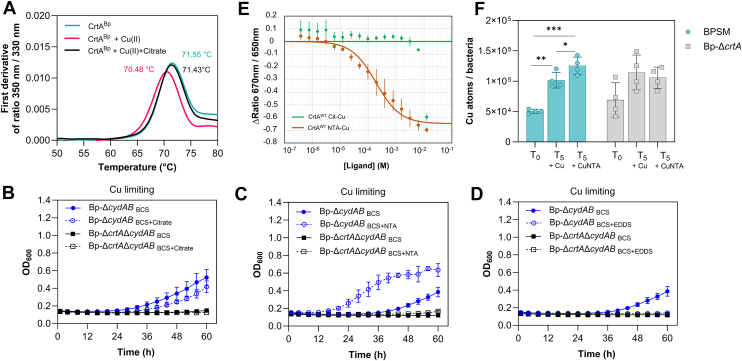
**Nitrilotriacetic acid (NTA) is a pseudoligand of CrtA^Bp^.***A,* citrate offsets the destabilizing effect of Cu(II) on CrtA^Bp^. Thermal unfolding profiles of purified CrtA^Bp^ were monitored by measuring its intrinsic Trp fluorescence using a Prometheus Panta apparatus (NanoTemper). The first derivative of the fluorescence emission ratio at 350 nm/330 nm (3 biological replicates) was plotted against temperature. The derivatives of the means of three melting curves of CrtA^Bp^ alone (*teal*), CrtA^Bp^ + 50 μM CuSO_4_ (*pink*), and CrtA^Bp^ + 50 μM CuSO_4_ + 100 μM citrate (*black*) are shown. *B–D, Bp*-Δ*cydAB* and *Bp-*Δ*crtA*Δ*cydAB* were grown with 20 μM BCS or 20 μM BCS + 500 μM citrate (*B*), 20 μM BCS + 500 μM nitrilotriacetic acid (NTA) (*C*), or 20 μM BCS + 500 μM ethylenediamine di-succinate (*D*). The BCS concentration was raised to 20 μM in these experiments to prolong the lag phase for the *Bp-ΔcydAB* strain and thus to enhance the potential effects of the ligands. Representative curves of three biological replicates are shown, with the means and SD calculated on three technical replicates. *E,* CrtA^Bp^ binding to NTA or NTA-Cu. Binding was measured through the relative change of fluorescence at 670 nm and 650 nm (spectral shift), with the amplitude of the signal between the apo and liganded proteins indicating the magnitude of the conformational change induced by ligand binding. The means and SD were calculated on three biological replicates. *F,* import of Cu after 5 min incubation of BPSM or *Bp*-Δ*crtA* with 2 μM CuSO_4_ or 2 μM CuSO_4_ + 500 μM NTA. The *Bp-cydAB* mutants could not be used here, because the bacteria were precultured with BCS to maximize *crtA* expression. In BPSM, copper levels increased significantly after Cu addition (*p* = 0.0047) and even more with Cu+NTA (*p* = 0.0003), with a modest but significant difference also observed between T5+Cu and T5+Cu-NTA (*p* = 0.0212), suggesting facilitated uptake in the presence of NTA. No significant differences were observed between the three conditions in *Bp-ΔcrtA*. Four biological replicates were used in this experiment, and statistical analysis was performed using Kruskal-Wallis one-way ANOVA followed by Conover's *post hoc* test for multiple comparisons of mean rank sums. Significant differences are shown: ∗, *p* < 0.05; ∗∗, *p* < 0.01; ∗∗∗, *p* < 0.001.

The absence of endogenous metallophore candidates suggested that in laboratory conditions CrtA^Bp^ might import Cu chelated by a metabolite. To test this hypothesis, we selected a number of small organic molecules capable of binding Cu(II) and potentially secreted by *B. pertussis* or present in the medium, and we performed thermal unfolding analyses of recombinant CrtA^Bp^ with copper-ligand complexes (Table S6). The Tm of CrtA^Bp^, 71.55 ± 0.04 °C, decreased to 70.48 ± 0.16 °C in the presence of 50 μM CuSO_4_. Copper is known to destabilize proteins and to cause their aggregation (4). A few compounds increased the Tm of CrtA^Bp^ in the presence of Cu, suggesting the formation of ternary complexes (Table S6). Citrate had the largest effect, reproducibly increasing the Tm by 0.95 ± 0.16 °C at 100 μM (Fig. 7*A*). This effect was specific as it was not observed for other compounds with higher affinities for Cu(II). Of note, citrate serves as a metallophore for Fe(III) uptake by the TBDT FecA (36).

### Identification of a pseudoligand

We tested the effect of citrate on growth of *Bp-ΔcydAB* in copper-limiting conditions but observed no improvement (Fig. 7*B*). As the synthetic Cu(II) chelator nitrilotriacetic acid (NTA) is structurally related to citrate and was reported to act like a siderophore for iron import in *P. aeruginosa* (37), it was also tested. NTA reproducibly improved *Bp-ΔcydAB* growth and decreased the lag phase, suggesting that CrtA^Bp^ could import a Cu(II)-NTA complex (Figs. 7*C* and S8). A natural Cu(II) chelator used as control, ethylenediamine di-succinate (EDDS) (38) instead impaired *Bp-ΔcydAB* growth (Fig. 7*D*), indicating that the effect of NTA is specific.

We compared the affinities of purified CrtA^Bp^ for Cu(II)-citrate and Cu(II)-NTA complexes using a ratiometric fluorescence assay. A K_d_ value around 200 μM was determined for the interaction of CrtA^Bp^ with NTA-Cu(II), whereas the affinities for NTA alone and for citrate-Cu(II) were millimolar (Fig. 7*E*). We also tested the CrtA_MM_ mutant protein in the same assay. Although an interaction with the NTA-Cu complex was detected, the binding curve reflected a different mode of binding than with the wt CrtA protein (Fig. S9). The growth phenotype of *Bp-C*rtA_MM_*ΔcydAB* in NTA-supplemented medium was also tested (Fig. S9). It was similar to that of *Bp-ΔcrtAΔcydAB,* most likely because the binding of NTA to the mutant protein is nonproductive *i.e*., unable to induce the conformational changes involved in transport.

Inductively coupled plasma mass spectrometry (ICP-MS) confirmed that NTA increased the uptake of Cu(II) by the parent strain BPSM but not by *Bp-ΔcrtA* (Fig. 7*F*). The reason for the modest but reproducible increase of the intracellular concentration of copper over 5 min might be the low affinity of CrtA^Bp^ for the Cu-NTA complex. In the absence of NTA, ICP-MS indicated that Cu(II) was likely imported by a nonspecific pathway in the two strains. Thus, the growth phenotypes, the fluorescence assay and the ICP-MS results indicated that NTA could be considered a pseudoligand of CrtA^Bp^. The affinity of CrtA^Bp^ for NTA-Cu(II), although lower than those of TBDT-ferrisiderophore complexes (32), appears to suffice to scavenge Cu(II) in the NTA-supplemented medium in laboratory conditions.

### Use of AlphaFold3 to identify ligand features

To sketch out the profile of *bona fide* CrtA^Bp^ ligands, AlphaFold (AF3) docking simulations (39) were carried out using structurally diverse compounds, including small carboxylic acids, polycarboxylic acid-type metallophores, other metallophores possibly present in *B*. *pertussis* niches, and hydroxamate- or catecholate-type siderophores as controls. We also included copper-chelating natural products (40), and Trojan-horse antibiotics to which *P. aeruginosa* was moderately sensitized by CrtA^Pa^ overexpression (25) (Table S7). We generated 25 models of CrtA^Bp^ in the presence of one Cu ion and one or two molecules of each compound (*i.e.*, Cu:ligand ratios of 1:1 or 1:2). The simulation produced docking results with iPTM scores > 0.6. The analysis pipeline is presented in Fig. S10. Ligands found within 5 Å of the experimental citrate binding site can be sorted into those binding at a 1:1 ratio and those binding at a 1:2 ratio (23 and two compounds in the binding site in > 70% of the models, respectively). All other compounds were not consistently found in the binding site at either ratio. Citrate, NTA, and EDDS were in the binding site at both 1:1 (>70% of the models) and 1:2 ratios (40%, 60% and 64% of the models, respectively). CYS and d-penicillamine were found there at the 1:2 ratio only (>70% of the models).

**Figure 8:**
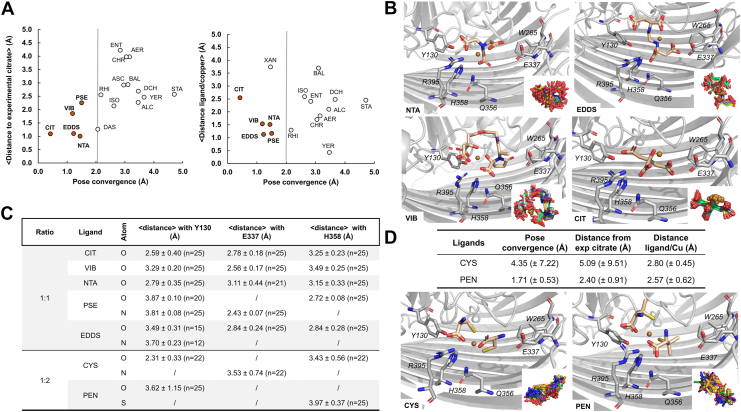
**Ligand profiling using AF3.***A,* analysis of docking results on 25 models per ligand (1:1 ratio). Ligands were first filtered according to their proximity (<5 Å) to the crystallographic binding site (23 ligands). The addition of the pose convergence criterion - positional consistency across models - allows removing six ligands (ACO, CEF, CHA, DFE, SF2 and XAN). The remaining ligands were filtered according to the distance between their center of mass and the copper ion. 15 and five ligands met all criteria with pose convergence values < 5 Å and < 2 Å, respectively. *B,* predicted Cu-ligand binding poses for NTA, citrate, vibrioferrin (VIB) and ethylenediamine di-succinate. *C,* average distances (<4 Å) between ligand donor atoms (N, O or S) and three key residues: Y130 (OH atom), E337 (Oε1 atom) and H358 (Nε2 atom). ‘n’ values indicate the number of models in which the interaction occurred at a distance shorter than 4 Å. *D,* analyses of the docking results on the models for CYS and penicillamine (1:2 ratio). In *panels* (*A* and *D*), the pose convergence values and the copper-ligand distances were calculated only for the models in which the ligands were in the binding cavity at the given ratio, while the average values for the distances between experimental and ligand positions are based on all 25 models. *Panels* (*B* and *D*) show a representative ligand positioning among the 25 models, with the insets displaying ligand positioning across the 25 models after protein superimposition.

To refine the selection, we filtered the results according to pose convergence *i.e.*, the consistency of ligand positioning across the 25 models, and to the distance between the ligand’s center of mass and the copper ion. At the 1:1 ratio, pose convergence cut-offs of 5 and 2 Å selected 15 and five Cu complexes, respectively, notably those with NTA, citrate, EDDS, vibrioferrin (VIB) and pseudopalin (PSE), all carboxylate-rich molecules (Fig. 8*A*). Nevertheless, not all carboxylate-containing compounds were stably positioned by AF3 in the ligand-binding cavity (*e.g.*, isocitrate or staphyloferrin), indicating some degree of specificity.

To pinpoint specific features of the productive CrtA^Bp^-NTA-Cu complex, we compared it with those formed with EDDS, VIB, PSE or citrate (Fig. 8, *B* and *C*). In all five cases, these ligands were at H-bonding distance from several conserved binding-site residues. The copper ion was coordinated by O atoms of the small molecule (VIB and CIT) or both O and N atoms (NTA and EDDS), but with unoccupied coordination site(s), notably oriented towards H358 in the NTA and VIB complexes (Fig. 8*C*). We probed the CrtA-NTA-Cu complex by building additional models after replacing the carboxylate groups or the N atom of NTA with -NH_2_ or -CH groups, respectively. These modifications decreased the convergence in the binding pocket and increased the distances with both Cu and the experimental position of citrate (Fig. S10). Finally, AF3 models of the ternary CrtA_MM_-NTA-Cu(II) complex showed a complete loss of ligand stabilization (Fig. S10).

At the 1:2 ratio, only CYS and penicillamine passed the 5-Å pose convergence cut-off. These small thiol-containing compounds provided four coordinating atoms, 2 S and 2 O, for the copper ion and were both positioned at H-bonding distance from several conserved binding-site residues (Fig. 8, *C* and *D*).

## Discussion

We showed here that CrtA^Bp^ is the prototype of a new TBDT subfamily involved in copper uptake and appears to be dedicated to providing copper for HCO-mediated respiration. CrtA contributes to bacterial fitness *in vivo*. We solved the structure of CrtA^Bp^ and identified a conserved ligand binding site, with in particular a His residue essential for function. Although ligand identification remains challenging for many TBDTs, we tried to define the profile of CrtA^Bp^ ligands. In laboratory conditions, the transporter seems to be selective for small Cu-binding organic molecules that notably harbor carboxylate groups. However, the *in vivo* ligand(s) of CrtA remain to be identified.

The strong conservation of CrtA among *B. pertussis* isolates supports its role in *Bordetella* biology. A growth phenotype of the deletion mutant was observed after inactivating the copper-independent oxidoreductase that sustains *B. pertussis* growth under copper restriction conditions. Loss of CydAB makes copper essential for aerobic respiration, and in these conditions the importance of CrtA^Bp^ indicated its role for HCO function. Of note, the HCO complexes were reported to be conditionally essential for *B. pertussis* in an animal model of infection (41). In *P. aeruginosa crtA*^*Pa*^ was strongly repressed in response to HCN, an endogenously produced poison of the HCOs (42). In contrast, HCN did not affect the expression of OprC, another Cu-importing TBDT in *P. aeruginosa*. This is consistent with CrtA^Pa^ having specialized in copper provision for HCOs also in that species. The presence of ScoI and PCuAC genes in other *crtA*-containing operons further suggests that this role might be shared by CrtA orthologs.

Contrary to OprC, reported to transport nonchelated Cu(I) (15), CrtA^Bp^ imports chelated Cu(II). The only two metallophores produced by *B. pertussis* were discarded as possible CrtA ligands. Nevertheless, CrtA^Bp^ allowed growth of *cydAB*-deficient bacteria under copper limitation in laboratory conditions, in the absence of any xenometallophore. This indicated that Cu(II) might be taken up in complex with carboxylate-harboring metabolite(s) present in the milieu. The *B. pertussis* culture medium contains millimolar concentrations of moderately affine copper binders, including CYS, glutathione, glutamate and other amino acids, which might be used by CrtA^Bp^ in the absence of alternatives. In the human airways the concentration of such metabolites increases upon *B. pertussis* infection due to the lytic activity of its toxins (19, 43).

In laboratory conditions, we identified NTA as a pseudoligand of CrtA^Bp^. NTA has a good affinity for Cu(II) thanks to its carboxylate groups and central N atom (44), and AF3 modeling suggested that it was among the best ligands among the compounds tested. The position of the His358 residue in the ligand-binding site could conceivably enable it to contribute to Cu chelation. Nevertheless, the affinity of CrtA for NTA-Cu is much lower than those between TBDTs and their *bona fide* ligands (45, 46), which most likely explains the need for high concentrations of NTA to enhance bacterial growth in copper-limiting conditions.

Given the apparent absence of specific endogenous metallophores for CrtA-type TBDTs, two nonmutually exclusive models may be proposed. It is possible that these transporters use xenometallophores present in the natural environments of the CrtA-producing bacteria. In the host, nutritional immunity might restrict the availability of copper, as recently described for *Mycobacterium tuberculosis* (7). To counter copper starvation, CrtA^Bp^ might import copper in complex with a xenometallophore produced by a species of the human respiratory tract microbiota. The use of a siderophore for copper uptake implies that Cu(II) outcompetes Fe(III) for chelation, which is conceivable given the top position of Cu(II) in the Irving-Williams series (47). Metallophores such as yersiniabactin have a broad metal selectivity (17). However, a mixture of siderophores including yersiniabactin induced *crtA* expression in *P. aeruginosa,* which rather indicates Cu starvation (48) and makes this metallophore an unlikely CrtA^Pa^ ligand candidate. Some other siderophores such as pyochelin can bind a variety of metals in addition to Fe(III), but nonferric complexes are taken up at low rates, implying that selectivity is mostly exerted at the transport step (45). Thus, Cu could be imported by CrtA-type TBDTs in complex with a xenometallophore only if productive interactions occur between the three partners.

An alternative model posits that ligand recruitment by CrtA^Bp^ might differ from the paradigm of TBDTs as highly selective transporters, CrtA being instead a ‘scavenger’ TBDT. Note that the high-selectivity concept no longer appears to hold true for all TBDTs anyway (31). Copper is required in much smaller amounts than iron by bacteria (49), except for specific, abundant cuproproteins (16). Consistently, few copper-importing TBDTs have been identified and in fewer species than iron-specific TBDTs. Another major difference is that Cu(II), the form available in oxygenated environments is soluble and therefore might not require high-affinity chelators, unlike insoluble Fe(III). Furthermore, given its strong affinity for organic molecules Cu(II) is necessarily complexed with ‘proximity ligands’ (47, 49). Together, these elements account for the possibility that CrtA^Bp^ has no high-affinity ligand but scavenges Cu chelated by small molecules with specific features, notably carboxylate groups, though not necessarily in a 1:1 Cu:ligand stoichiometry. By providing a coordination of the metal in the ternary complex, the essential His residue of CrtA might serve as a selectivity filter ensuring that only Cu(II) complexes are imported. An additional filter might be the interactions with plug residues necessary to activate TonB-dependent transport.

Studies on Trojan-horse metallophore-antibiotic complexes have suggested that CrtA^Pa^ might transport several of those, albeit inefficiently (25), although it remains to be seen if these molecules form complexes with copper. Other TBDTs of *P. aeruginosa* also appear to take up these Trojan-Horse compounds (25, 50), suggesting that they too might be more promiscuous than usually considered.

## Experimental procedures

### *In silico* analyses

We collected TBDTs from the nonredundant NCBI database (1/1/2025) using the TonB_dep_Rec_b-barrel.hmm profile (https://www.ebi.ac.uk/interpro/entry/pfam/PF00593/), reduced sequence redundancy to 70% using CD-HIT (http://bioinformatics.org/cd-hit/), selected a 500 to 1200 residues size range and subjected the remaining 297,770 proteins to sequence similarity network analyses using the Enzyme Function Initiative Enzyme Similarity Tool (EFI-EST) (https://efi.igb.illinois.edu/efi-est/). Networks were visualized by Cytoscape using the yFiles Organic Layout (51). To further analyze the CrtA subfamily, all orthologs of CrtA^Bp^ were collected, and the corresponding bacterial genomes were downloaded on a local server. Rodeo-type analyses were performed to extract the genetic environment of *crtA* genes (21), and the genomes were searched for co-occurrence of other genes of interest. An antiSMASH analysis (https://antismash.secondarymetabolites.org/) was also performed to identify frequent secondary metabolite gene clusters in these genomes.

### Bacterial strains, plasmids and growth conditions

*B*. *pertussis* strains were grown on Bordet-Gengou (BG) blood-agar (48 h, 37 °C), then precultured for 24 h in Stainer-Scholte (SS) medium (initial OD_600_ = 0.25) at 37 °C. For growth assays, SS medium was supplemented with 20 mM MgSO_4_ unless otherwise indicated, as we found out that this condition maximizes expression of the operon. BCS was added to 50 μM for BPSM and *Bp-ΔcrtA*, 15 μM for *Bp-ΔcydAB* strains or 20 μM to prolong the lag phase. Cultures were inoculated into 96-well plates (initial OD_600_ = 0.15) and grown at 37 °C under agitation for 60 h using an EnSight Multimode plate reader (PerkinElmer). CuSO_4_ was added at 2 μM where indicated. Streptomycin and gentamicin were added at 100 and 10 μg/ml, respectively. In-frame deletions of *crtA*, *cydAB*, *bp2930-bp2933 (cyoABCD)*, *bp3740-bp3744 (ctaCDFEG), bp2921* and *bp2924 (bufA1)* were generated by cloning their PCR-amplified flanking regions into pSS1129 (52). Recombinant pSS1129 plasmids were used to transform *Escherichia coli* SM10 to perform allelic exchange by conjugation with BPSM or its derivatives. Genome-wide sequencing of BPSM-Δ*crtA*, BPSM-Δ*cydAB* and BPSM-Δ*crtA*Δ*cydAB* was performed to rule out extraneous mutations. H358S was introduced by site-directed mutagenesis, and for *Bp*-*crtA*_MM_ synthetic fragments (GeneCust) were used. The *bufA1* mutant was generated by cloning a synthetic gene with a stop codon at Met8 into pSS1129. The *bp3224 to 3225* operon encoding a second cytochrome bd ubiquinol oxidase (not expressed in laboratory conditions) was also inactivated in all strains. Knockouts mutants of *alcA* and *bp3224-2*5 were constructed by interrupting the genes with a suicide plasmid. The expression plasmid of *crtA*^*Bp*^, pT7K-BfrG-twstrep, was constructed by inserting the XbaI-HindIII fragment of pT7-prnCt-Stag (53) into pET24 d (Invitrogen), adding a twin-strep tag sequence in the BamHI site and cloning PCR-amplified *crtA*^Bp^ in BamHI-HindIII. The mutant version of *crtA*^Bp^ was similarly introduced in the expression plasmid. For complementation, the natural regulation system involves a CruR- and Rho-dependent post-transcriptional regulation that could not be reproduced on a plasmid, hence we used a Plac promoter in a low-copy plasmid. The 5′ moiety of *crtA*^Bp^ was PCR-amplified, restricted with KpnI and XhoI and used in a three-way ligation with the XhoI-HindIII fragment of pT7K-BfrG-twstrep and KpnI-HindIII restricted pBBR1-MCS5 (54).

### Protein expression and purification

*E. coli* BL21(DE3)-omp5 (55) carrying pT7K-BfrG-twstrep (wt or MM version) was grown in LB at 37 °C until OD_600_ = 0.75 and treated with 1 mM IPTG for 3 h. Cells were harvested, resuspended in lysis buffer (50 mM sodium phosphate pH 7, 300 mM sodium chloride, 0.01 mg/ml DNase, and protease inhibitors (Complete, Roche)) and lysed using a French press. The clarified lysate was ultracentrifuged (100,000*g*, 1 h, 10 °C). Membrane proteins were extracted sequentially with 0.8% and 1.5% βOG in 50 mM sodium phosphate pH 8, 150 mM NaCl, for 1 h at 30 °C. The second extract was loaded on a 1-ml StrepTrap HP column in this buffer with 1% βOG, and CrtA^Bp^ was eluted with 50 mM biotin in the buffer. CrtA^Bp^-containing fractions were concentrated by ultrafiltration (50K, Amicon Ultra) and buffer-exchanged to 50 mM Hepes pH 8, 150 mM NaCl, 1% βOG.

### Crystallization and X-ray structure determination

CrtA^Bp^ (10 mg/ml) was subjected to crystallization screens (JCSG+, Stura FootPrint, MEMGold2 ECO) by mixing 0.7 μl protein + 0.7 μl reservoir solution in a sitting-drop setting, using a TECAN FreedomEVO pipetting robot. Crystals appeared within days. Useful crystals were obtained in 20 mM NaCl, 50 mM MES (pH 5.5), 14% PEG350 MME (form 1) and in 0.1 M Na-Citrate (pH 5.5), 16% PEG4K, 10% isopropanol (form 2). Crystals were soaked in their respective mother liquor + 15% glycerol, mounted on litholoops and directly frozen in liquid nitrogen before evaluation of diffraction quality at the ESRF ID30 B beamline (ESRF, Grenoble). Structures were solved by molecular replacement (Molrep) (56) using an AF3 CrtA^Bp^ model (39) and refined with Buster, from Global Phasing Limited, with the TLS refinement option.

### Ligand screening by thermal unfolding analysis of CrtA^Bp^

The samples were prepared in a final volume of 10 μl with purified CrtA^Bp^ (6 μM) alone or with each of the metabolites (50 μM) and CuSO_4_ (50 μM) in 50 mM Hepes (pH 8), 150 mM NaCl, 1% βOG. Mixtures were loaded into microcapillaries which were placed in Prometheus Panta instrument (NanoTemper Technologies). Thermal unfolding was recorded from 25 °C to 95 °C at a rate of 2 °C/min by measuring intrinsic tryptophan fluorescence (excitation 280 nm, emission 330 and 350 nm). The first derivative of the 350 nm/330 nm fluorescence ratio was plotted using GraphPad Prism v10.

### Affinity measurements by ratiometric fluorescence assay

Purified CrtA^Bp^ (wt and MM) proteins were labeled with fluorescent dye RED-NHS (NanoTemper) and incubated in 50 mM Hepes (pH 8.0), 150 mM NaCl, 1% βOG for 30 min at room temperature in the dark. Initial binding between CrtA^Bp^ and each ligand was verified prior to affinity measurements *i.e.*, 40 nM of protein was mixed with an equal ligand concentration. For titration, labeled CrtA^Bp^ (20 nM) was mixed with serially diluted ligands. Binding was measured using the Monolith X instrument (NanoTemper), and the spectral shift between 670 nm and 650 nm was analyzed with MO.Control software v2.5.4 (NanoTemper). All experiments were performed in triplicates.

### AF3 modeling

The smiles of the compounds in their predicted protonation state (pH 7) were generated by the Marvin-Sketch program. Residues 37 to 732 of CrtA^Bp^ were used to build the model. Surface His residues were modified to Gln to avoid nonrelevant copper binding. For each simulation, a Cu(II) atom was independently added to one or two copies of the ligand. At the 1:2 ratio model analyses focused on 16 small compounds unable to provide full coordination to the metal. Calculations were performed on the Hercules2 and the Lyra clusters (Consortium des Equipements en Calcul Intensif), for the GPU-based model inference using NVidiaA40 or NVidia A6000GPU. Five randomly selected seed numbers were used, generating at least 25 models per model inference. Docking results were filtered by two performance metrics: the pose convergence (Å), which represents convergence of docking results measured by mean pairwise root mean square deviation across generated models with the DockRMSD program (57); and the distances (Å) measured between the ligand’s center of mass and that of citrate in the reference structure, and between the ligand’s center of mass and the position of the Cu ion.

### Intracellular copper quantification

Four precultures of BPSM and Bp-Δ*crtA* were grown in SS medium with 50 μM BCS until OD_600_ ≈ 2 and used to inoculate cultures at OD_600_ = 0.1 in SS medium with 20 mM MgSO_4_ and 20 μM BCS. After 20 h, cells were harvested by centrifugation, washed in prewarmed SS medium and resuspended in Chelex-treated SS medium. For T_0_, 8-ml suspensions were mixed with ice-cold stop solution (PBS, 5 mM BCS, 5 mM Trien, a Cu(II) chelator) and placed on ice. For T5, 2 μM CuSO_4_ or 2 μM CuSO_4_ + 500 μM NTA (preformed complex) were added to bacteria, and the stop solution was added after a 5-min incubation. Bacteria were centrifuged (10,000×*g*, 10 min, 4 °C), washed twice with ice-cold PBS + 5 mM Trien, dried (99 °C, 3 h), resuspended in 700 μl 65% nitric acid and incubated at 80 °C overnight. After cooling, 6.3 ml of Chelex-treated water was added and samples were filtered (0.45 μm). ^63^Cu levels were measured using a single quadrupole mass ICP-MS 7850 spectrometer (Agilent) with He as collision gas and Kinetic Energy Discrimination to eliminate spectral interferences. An external calibration curve was performed using a 100 mg/L Cu standard solution in 5% HNO_3_ (Analytichem) and ^74^Ge as internal standard. All solutions were prepared in ultrapure Milli-Q water and acidified at 2% with nitric acid (67–69%, ultrapure trace metal grade). Statistical analyses were performed as described (58).

## Statistical analyses

Statistical analyses were performed where required. For the animal experiments, statistical significance was determined using a nonparametric permutation-based ANOVA (∗, *p* < 0.05; ∗∗∗, *p* < 0.001). For the ICP-MS experiment, statistical analysis was performed using Kruskal-Wallis one-way ANOVA followed by Conover's *post hoc* test for multiple comparisons of mean rank sums (∗, *p* < 0.05; ∗∗, *p* < 0.01; ∗∗∗, *p* < 0.001).

### Other experiments

For immunoblot analyses, *B. pertussis* cultures (10 ml, OD_600_ ≈ 1.5) grown in SS medium + 50 μM BCS were lysed in 50 mM Tris-HCl (pH 8) using a Ribolyser (speed 6, 50 s, four cycles). The clarified lysates were ultracentrifuged (100,000*g*, 1 h, 4 °C) to isolate membranes. After SDS-PAGE to separate the proteins and transfer to nitrocellulose, CrtA^Bp^ was detected using a guinea pig polyclonal antibody raised against the recombinant protein (1:2500) and anti–guinea pig–HRP (1:5000). Detection was performed with the Amersham ECL Prime Western Blotting System using the Amersham Imager 600 (GE). For RT-qPCR, 8 ml of *B. pertussis* liquid cultures with 50 μM BCS were harvested at OD_600_=1 by centrifugation at 4 °C after adding 2 ml of a phenol:ethanol solution (5:95, v/v). Total RNA was extracted using Tri-Reagent (Invitrogen), followed by a DNAse I treatment (Sigma Aldrich). Reverse transcription was performed with the Verso cDNA Synthesis Kit (Thermo Fisher Scientific). qPCR was carried out on a Roche LightCycler 480 Instrument II using the Takyon Low-ROX SYBR kit (Eurogentec). The experiments were conducted with biological replicates and three technical replicates, and *bp2921* expression levels were normalized to those of the housekeeping gene *bp3416*. Single-strain experimental infections of mice were performed as described (21). For the co-infection experiment BPSM and *Bp-DcrtA*^*Bp*^ were grown in liquid SS medium supplemented with BCS to prevent intracellular copper accumulation and mixed in equal numbers (5 × 10^4^ of each strain in 20 ml) for nasal inoculation. The two strains were differentiated by their antibiotic resistance markers (59). The study protocol was approved by the Ethical Committees of the Region Nord-Pas-de-Calais and the Ministry of Research under the agreement number APAFIS #51236 to 2024041114331031 v7.

## Data availability

This article contains supporting information. All relevant data are within the article and its supporting information files. The raw data can be shared upon request to francoise.jacob@crns.fr. This article contains X-ray crystallographic data. The structures were deposited in the RCSB database (pdb 9RVQ and 9RVX).

## Supporting information

This article contains supporting information (60, 61, 62, 63, 64, 65, 66, 67, 68, 69, 70, 71, 72).

## Conflict of interest

The authors declare that they have no conflicts of interest with the contents of this article

## Supporting information

Hachmi Supporting Information
