# Supplementary material for: A new family of TonB-dependent copper transporters linked to respiratory oxidase function
Source: J Biol Chem. 2026 Jan 20;302(3):111180. doi: 10.1016/j.jbc.2026.111180 (PMC12914433; doi:10.1016/j.jbc.2026.111180)
Supplement: Hachmi Supporting Information [file mmc1.docx]

*Supporting information for the manuscript*

### A new family of TonB-dependent copper transporters

**linked to respiratory oxidase function**

M. Hachmi^1^, M. Mirgaux^2^, R. Wintjens^2^, C. Carassus^3^, P. Arnoux^3^, G. Roy^1#^, A. Rivera-Millot^1#^, S. Slupek^1^, A.S. Debrie^1^, V. Alaimo^4^, G. Billon^4^, L. Coutte^1^, R. Antoine^1*^, F. Jacob-Dubuisson^1*^

This file contains

- 10 supporting figures
- 8 supporting tables

**A**


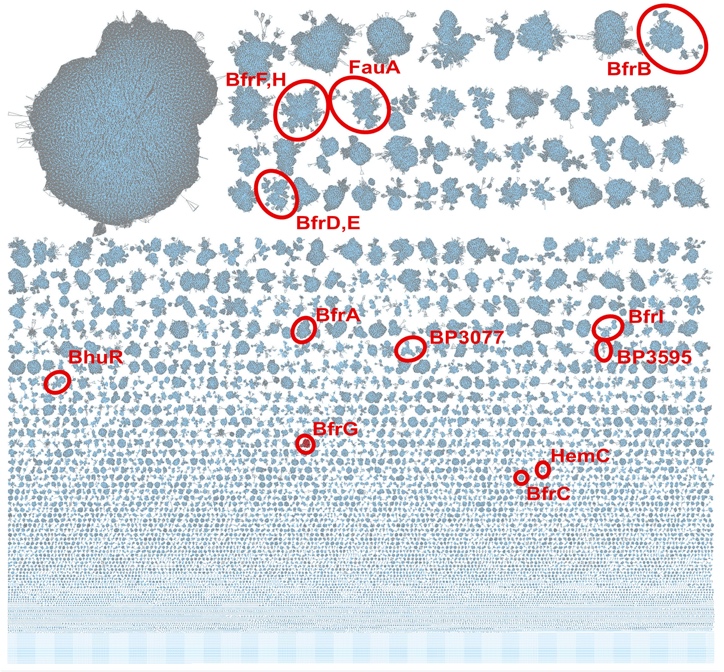


**B**


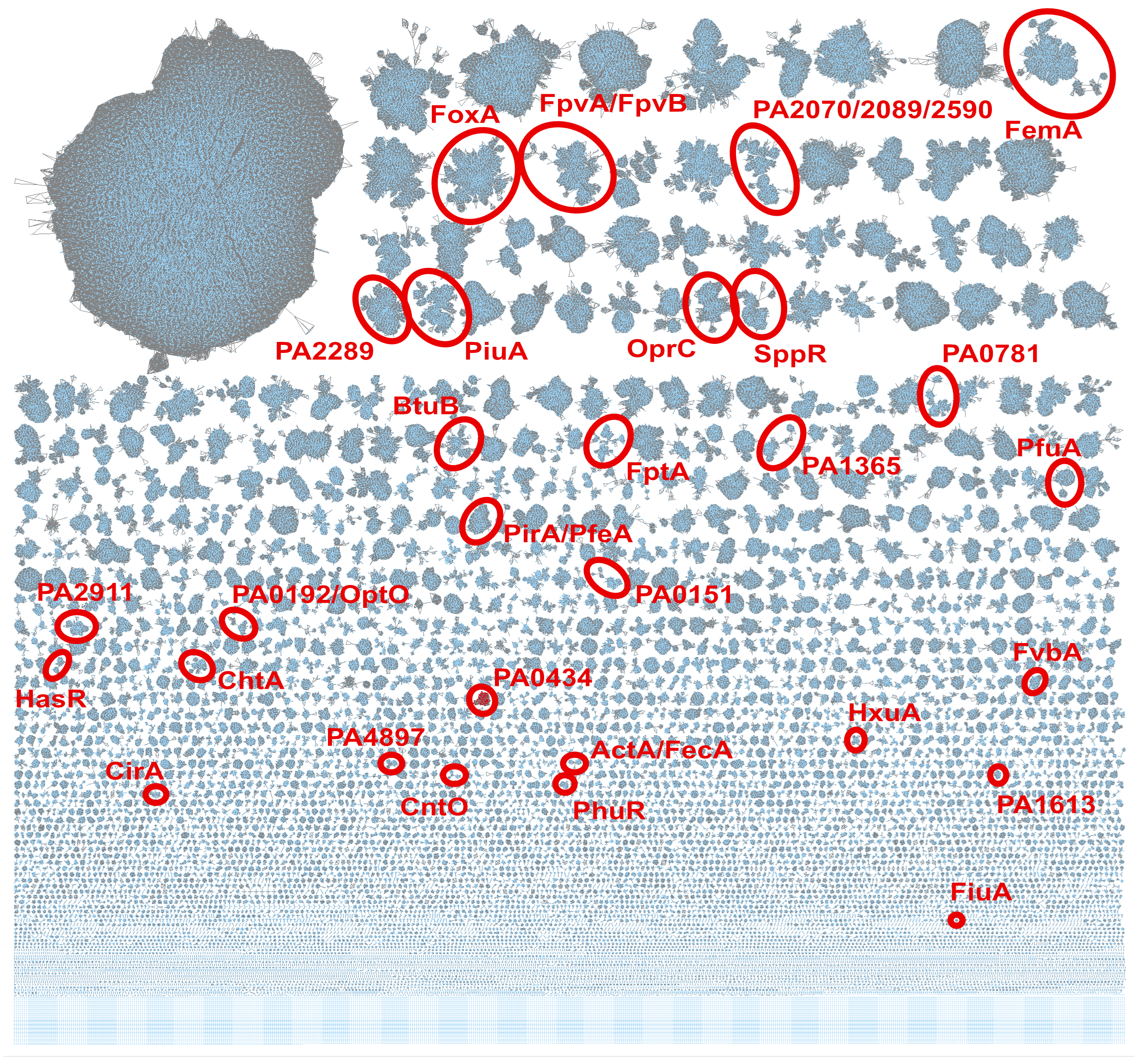


**C**

**
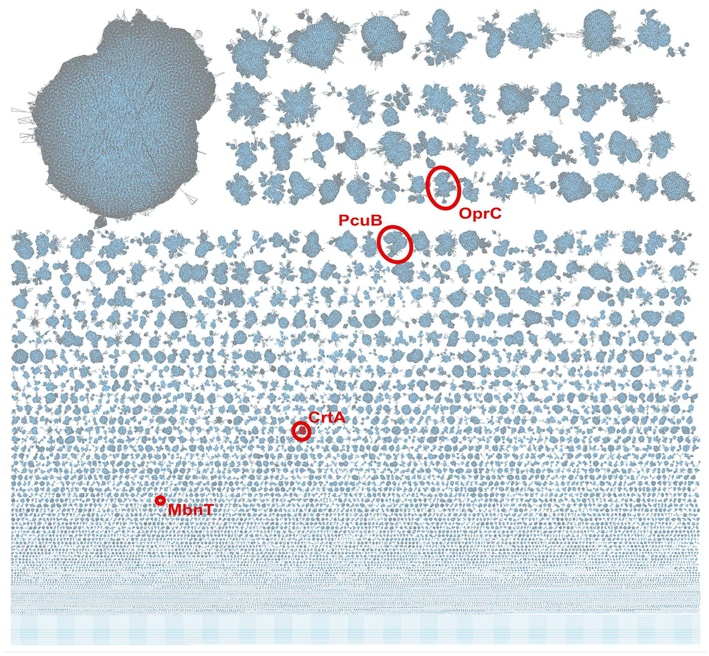
**

**Figure S1. TBDTs of *B. pertussis* and *P. aeruginosa* and copper-importing TBDTs.** All TBDTs of *B.* *pertussis Tohama I* (**A**) and *P. aeruginosa* PA01 (**B**) were positioned on the sequence similarity node network shown in Fig. 1. **C**, TBDTs currently known to import Cu were positioned on the same node network, yielding four sequence clusters. The OprC cluster also contains NosA of *Pseudomonas stutzeri,* which is required for the activity of the cuproprotein N_2_O reductase (61). Another copper starvation-inducible TBDT, PcuB of *Bradyrhizobium japonicum* (62) belongs to a cluster distinct from both those of OprC and of CrtA^Bp^ and PA0434. Similarly, the TBDT MbnT (63) of the model methanotroph *Methylosinus trichosporium* is yet in a different SSN cluster. The Genbank ID numbers of all the proteins found in the labeled sequence clusters are provided in SI Table S2.


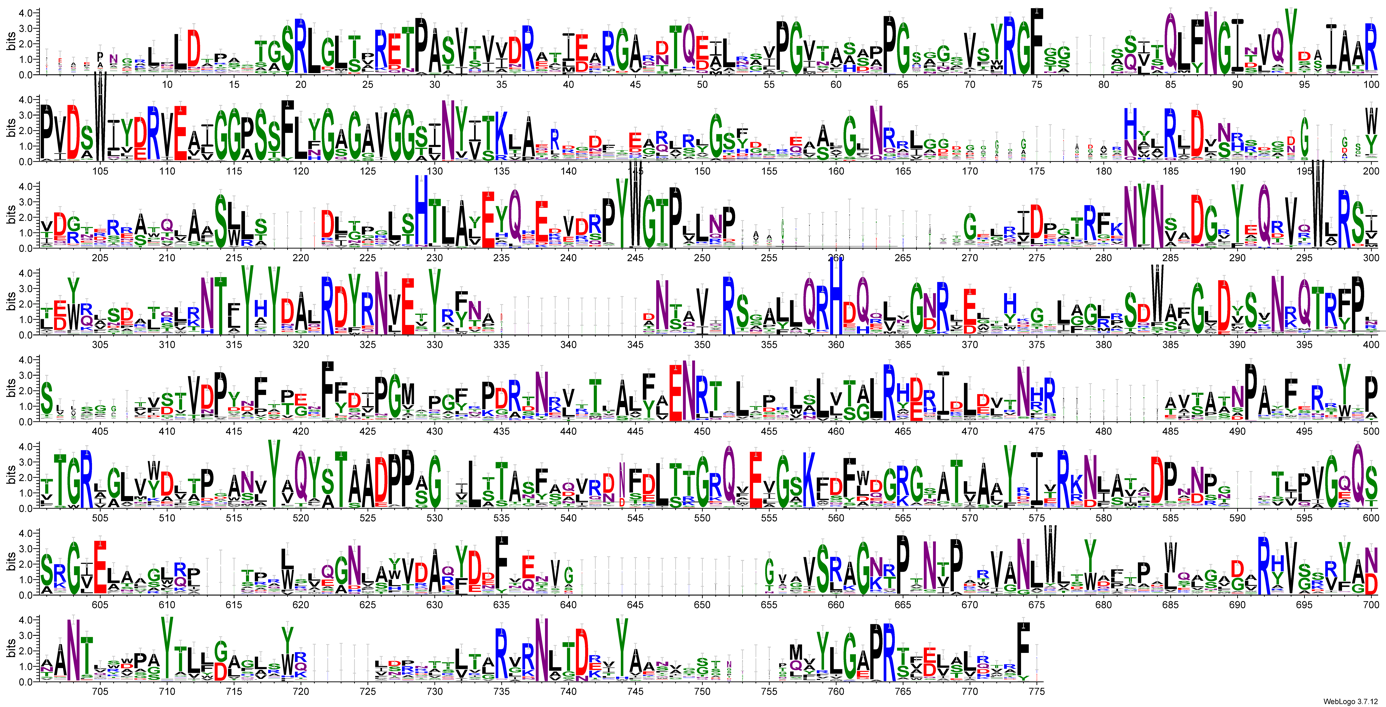


**Figure S2. Sequence logo of the CrtA family.** The two conserved His residues are highlighted, and the conserved residues of the ligand binding cavity are boxed (see below).

**
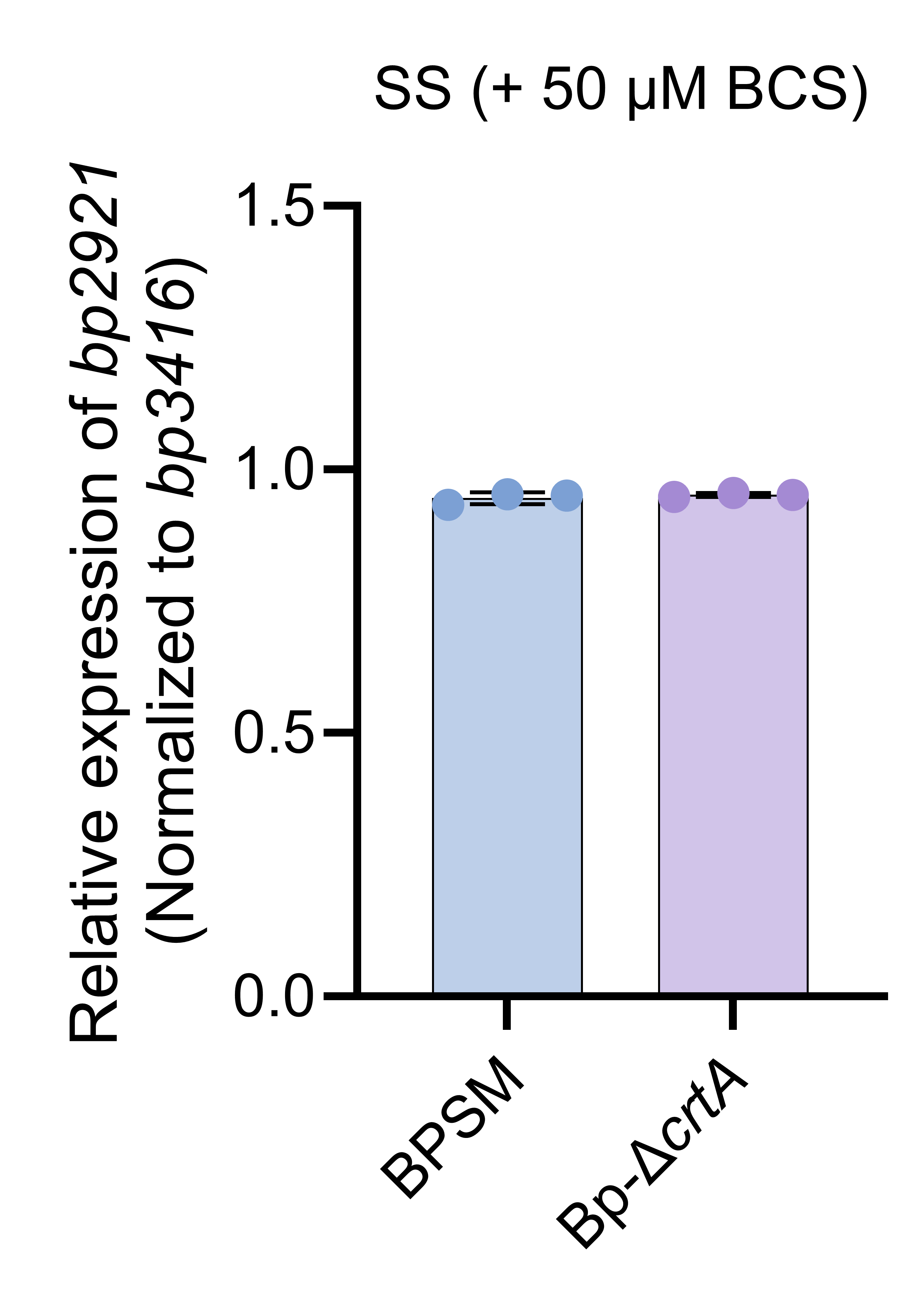
**

**Figure S3. Reverse-transcription-quantitative PCR (RT-qPCR) analysis of *bp2921.*** The *crtA^Bp^* gene is part of the *cruR-crtA-bp2921* three-gene operon (22). RT-qPCR analyses were performed to show that the in-frame deletion of *crtA* did not affect the level of expression of *bp2921*. The strains were grown with 50 μM BCS. The RT-qPCR data on *bp2921* in BPSM and *Bp-ΔcrtA* were normalized to the expression levels of *bp3416*, a housekeeping gene unaffected by the presence of Cu in the medium.

**A B**

**
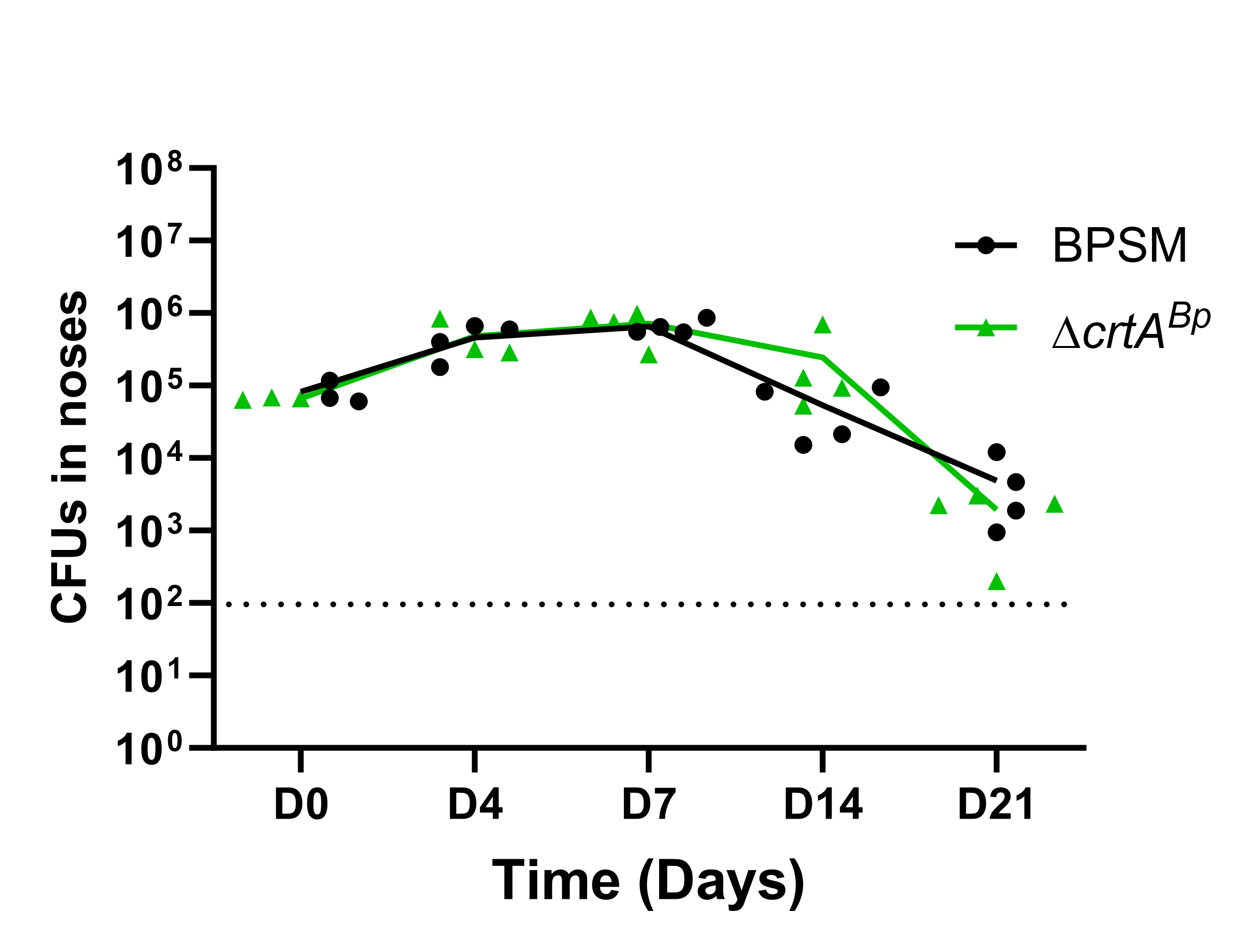

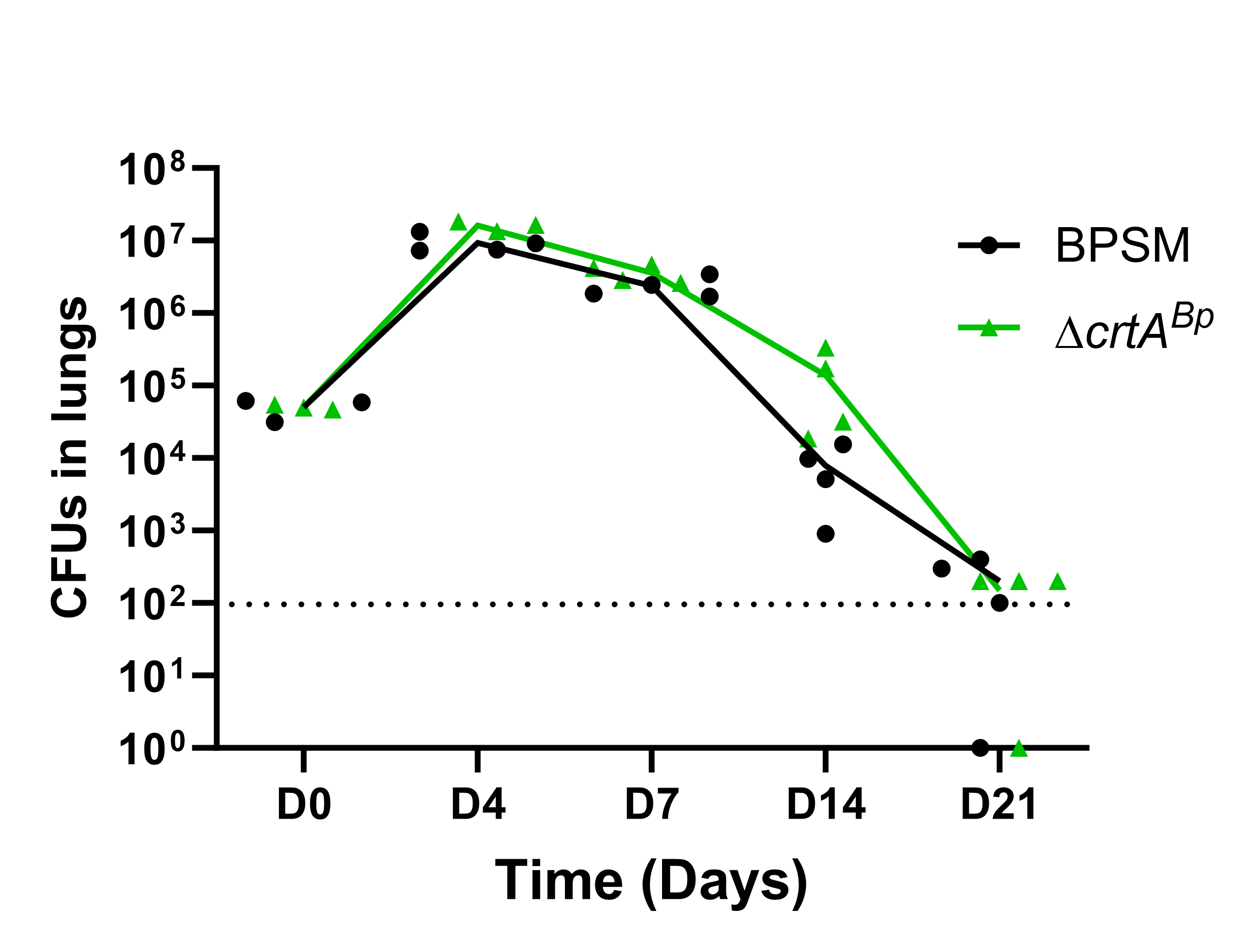
**

**Figure S4. *In vivo* experiment with *crtA* mutant**. Colonization of mouse nasal cavities (**A**) and lungs (**B**) following intranasal infection with the wt *B. pertussis* strain BPSM (black circles) or the *Bp-*∆*crtA* mutant (green triangles). Each point represents the bacterial load recovered from an individual organ of a single mouse. Solid lines connect the geometric means of bacterial counts for each group. The dotted lines represent the limits of detection.

**
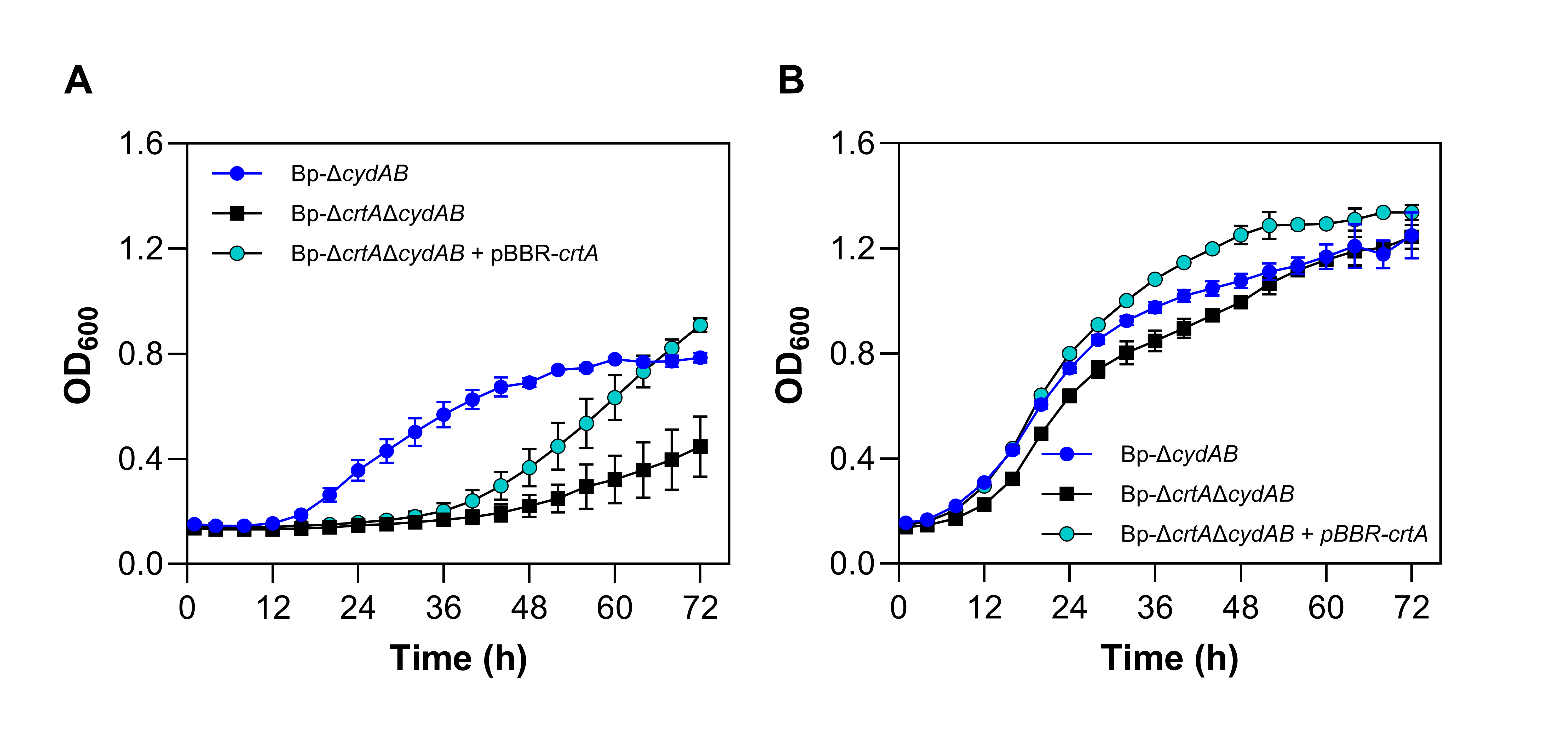
**

**Figure S5.** **Complementation of the growth phenotype of *Bp-ΔcrtAΔcydAB***. The low-copy plasmid pBBR1-MCS5 harboring *crtA* under the control of a *lac* promoter was used to complement the mutant strain. Growth was monitored in SS medium containing 15 μM BCS (copper-limiting condition, **A**) or with 2 μM M CuSO_4_ (copper-replete conditions, **B**). Although the complemented strain grew faster than *Bp-ΔcrtAΔcydAB*, its growth curve did not reproduce that of *Bp-ΔcydAB.* The longer lag might be explained by the time needed to accumulate enough CrtA in the complemented strain.

**
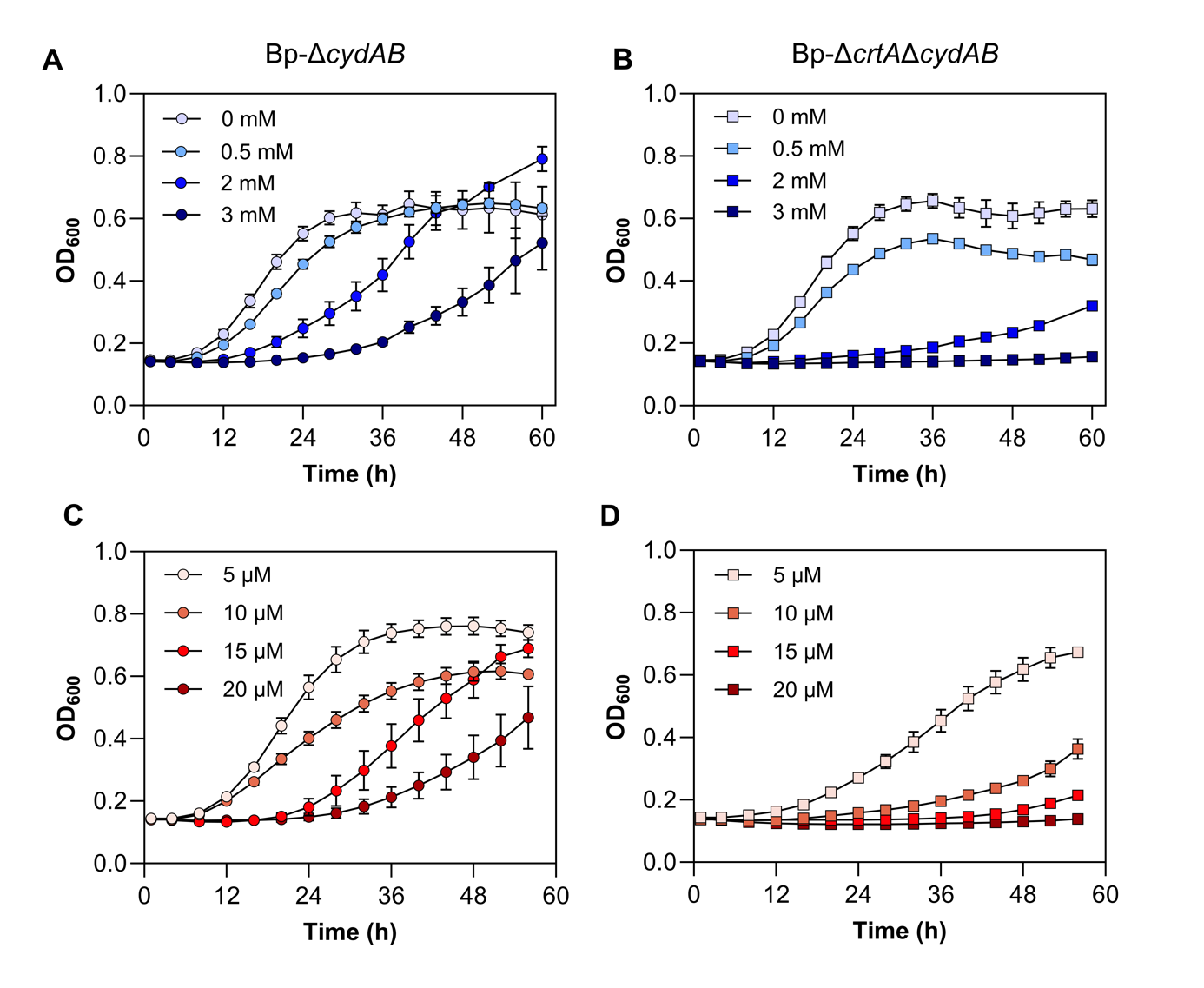
**

**Figure S6. Cu(II) availability modulates the growth of *Bp-cydAB* mutant strains under copper-limiting conditions.** **A** and **B**, Growth of *Bp-∆cydAB* (**A**) and *Bp-∆crtA∆cydAB* (**B**) in SS medium supplemented with 15 µM BCS and containing various concentrations of ascorbate (0, 0.5, 2 or 3 mM, final concentrations). In the SS medium ascorbate is present at 2 mM. Ascorbate reduces Cu(II) to Cu(I), which is chelated by BCS. Hence lower concentrations of ascorbate increase the availability of Cu(II), and the lag phase shortens. **C** and **D**, growth of *Bp-∆cydAB* (**C**) and *Bp-∆crtA∆cydAB* (**D**) in SS medium supplemented with increasing BCS concentrations (5, 10, 15, or 20 µM). Higher BCS concentrations prolonged the lag phase, consistent with decreased copper availability. Growth of *Bp*-∆*crtA*∆*cydAB* was consistently slower than that of *Bp*-∆*cydAB*, indicating that Cu(II) is imported by CrtA. The improved growth of *Bp*-∆*crtA*∆*cydAB* under conditions of low BCS and ascorbate concentrations indicates that in those conditions Cu(II) enters through another pathway, probably porins. The curves shown are representative of three biological replicates. The means and SD were calculated on 4 technical replicates.


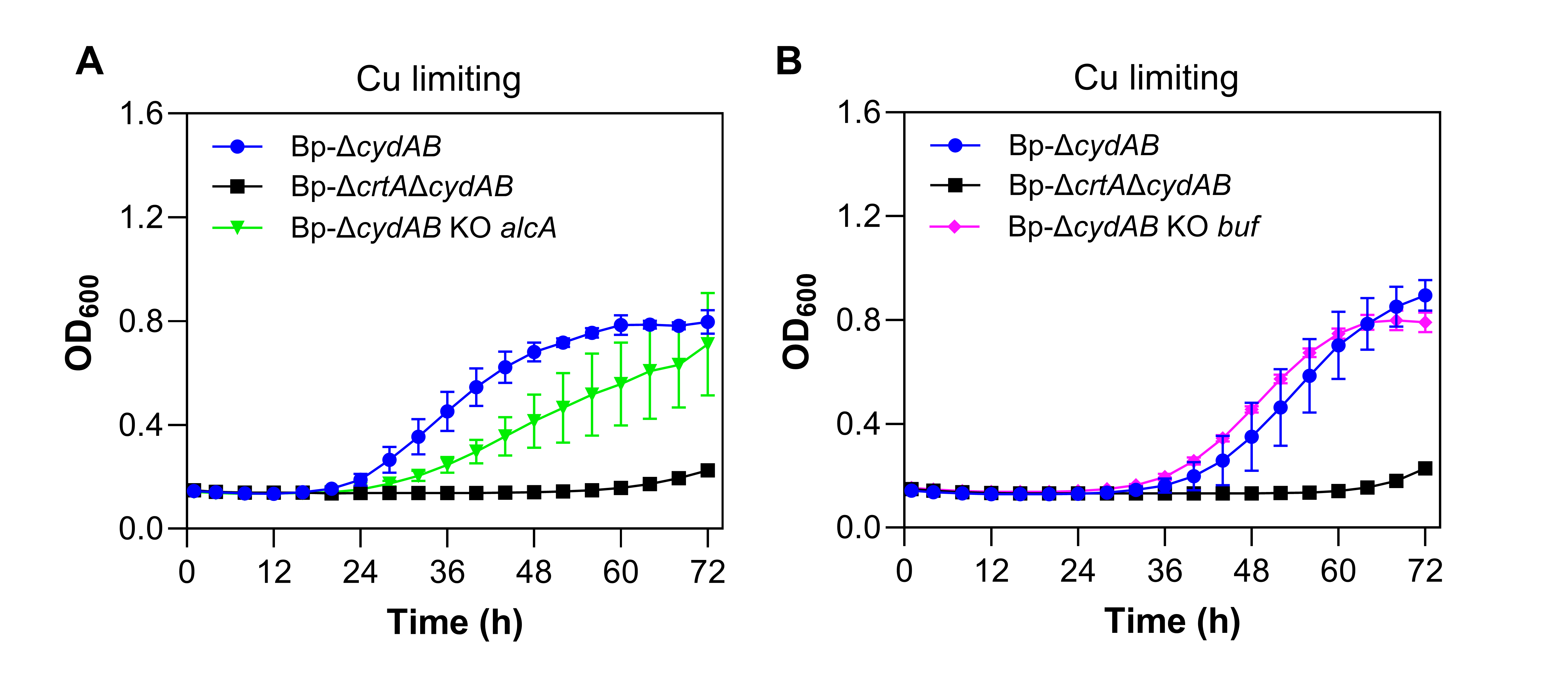


**Figure S7. Alcaligin and bufferin are not required for CrtA-mediated copper acquisition in *B. pertussis*.** **A**, growth of *Bp-*∆*cydAB*, *Bp-*∆*crtA*∆*cydAB* and *Bp-*∆*cydAB-KOalcA* (which does not produce alcaligin) in SS medium supplemented with 15 µM BCS. **B**, growth of *Bp-*∆*cydAB*, *Bp-*∆*cydAB*∆*crtA* and *Bp-*∆*cydAB-KObuf* (which does not produce bufferin) in the same conditions except that no MgSO_4_ was added to the medium (Bvg^+^ conditions) to maximize expression of the bufferin operon. The results are representative of three biological replicates. Neither the alcaligin-deficient nor the bufferin-deficient strains phenocopied the CrtA-deficient strain *Bp-*∆*crtA*∆*cydAB*, indicating that these metallophores are not involved in CrtA-dependent copper uptake. The intermediate growth of *Bp-*∆*cydAB*-*KOalcA* is most likely explained by the limited iron uptake of this strain due to the absence of the only siderophore produced by *B. pertussis*.

**
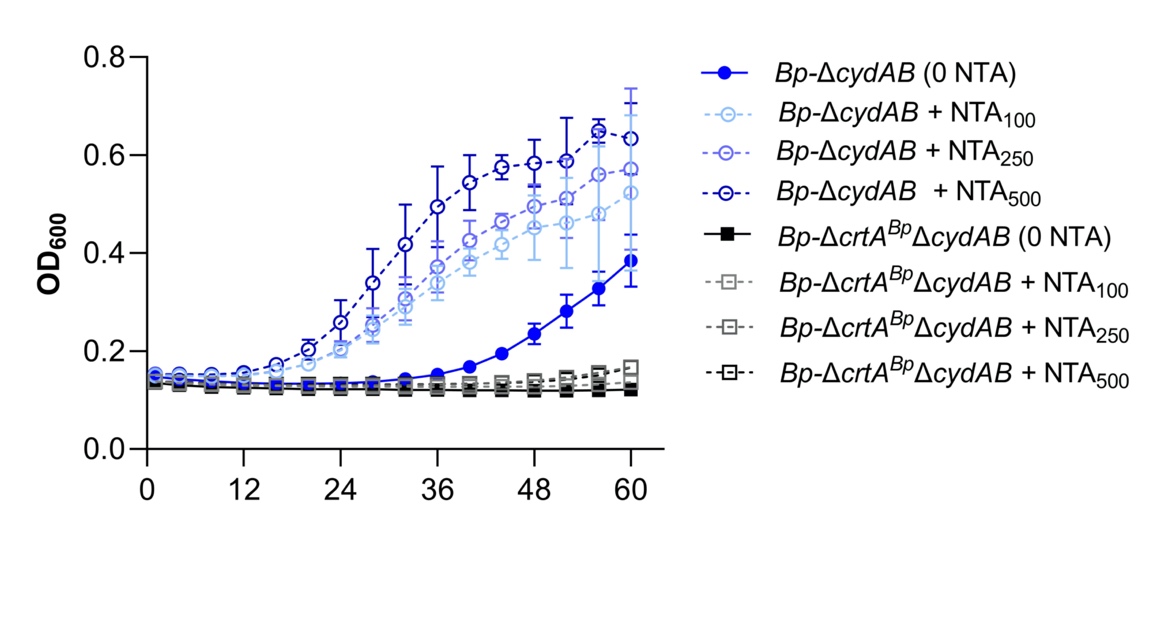
**

**Figure S8. Growth of *Bp-*∆*cydAB* and *Bp-∆crtA*∆*cydAB* in the presence of increasing NTA concentrations.** The cultures were performed with 20 μM BCS + the indicated concentrations (0 to 500 μM) of NTA. For the CrtA producing strain *Bp-*∆*cydAB* (blue symbols)*,* increasing the concentration of NTA improved growth, unlike for *Bp-∆crtA∆cydAB* (black symbols).

**
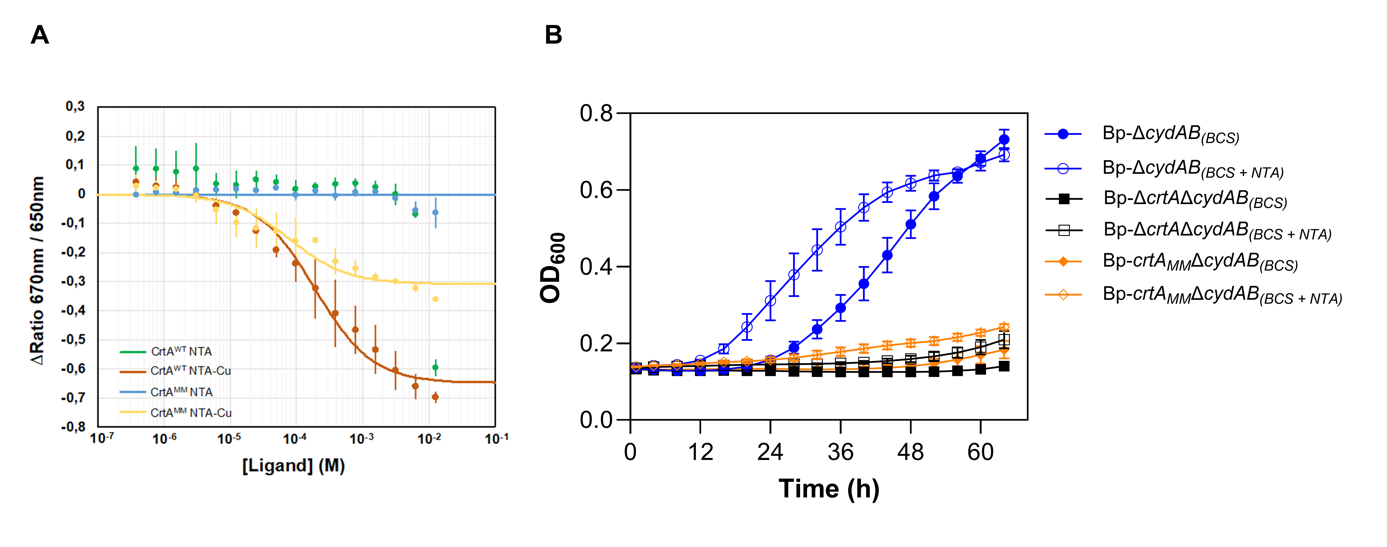
**

**Figure S9. Control assays.** Binding of the indicated molecules or complexes to CrtA^Bp^ was measured through the relative change of fluorescence of the protein at 670 nm and 650 nm (spectral shift). In **A**, the binding of NTA or the NTA-Cu complex to CrtA or CrtA^MM^ were compared. The blue and green curves (NTA alone) are superimposed and represent non-binders. The distinct plateaus reached for the yellow and orange curves show that the nature of NTA-Cu binding differs between the two proteins. Although NTA-Cu bound to CrtA^MM^, the conformational changes induced were different from that induced in the wt transporter. The data shown are representative of three independent experiments and the errors are calculated as SD. This figure complements Fig. 7E. **B**, *Bp*-∆*cydAB*, *Bp-*∆*crtA*∆*cydAB* and *Bp-crtA_MM_*∆*cydAB* were grown with 20 µM BCS or 20 µM BCS + 500 µM NTA. Representative curves of three biological replicates are shown, with the means and SD calculated on three technical replicates. Given that transport is a complex process involving recognition, structural change of the plug, and motions of the extracellular loops and the plug, we conclude that the altered mode of binding of NTA-Cu explains the absence of import activity by CrtA^MM^ as seen in the growth assays.


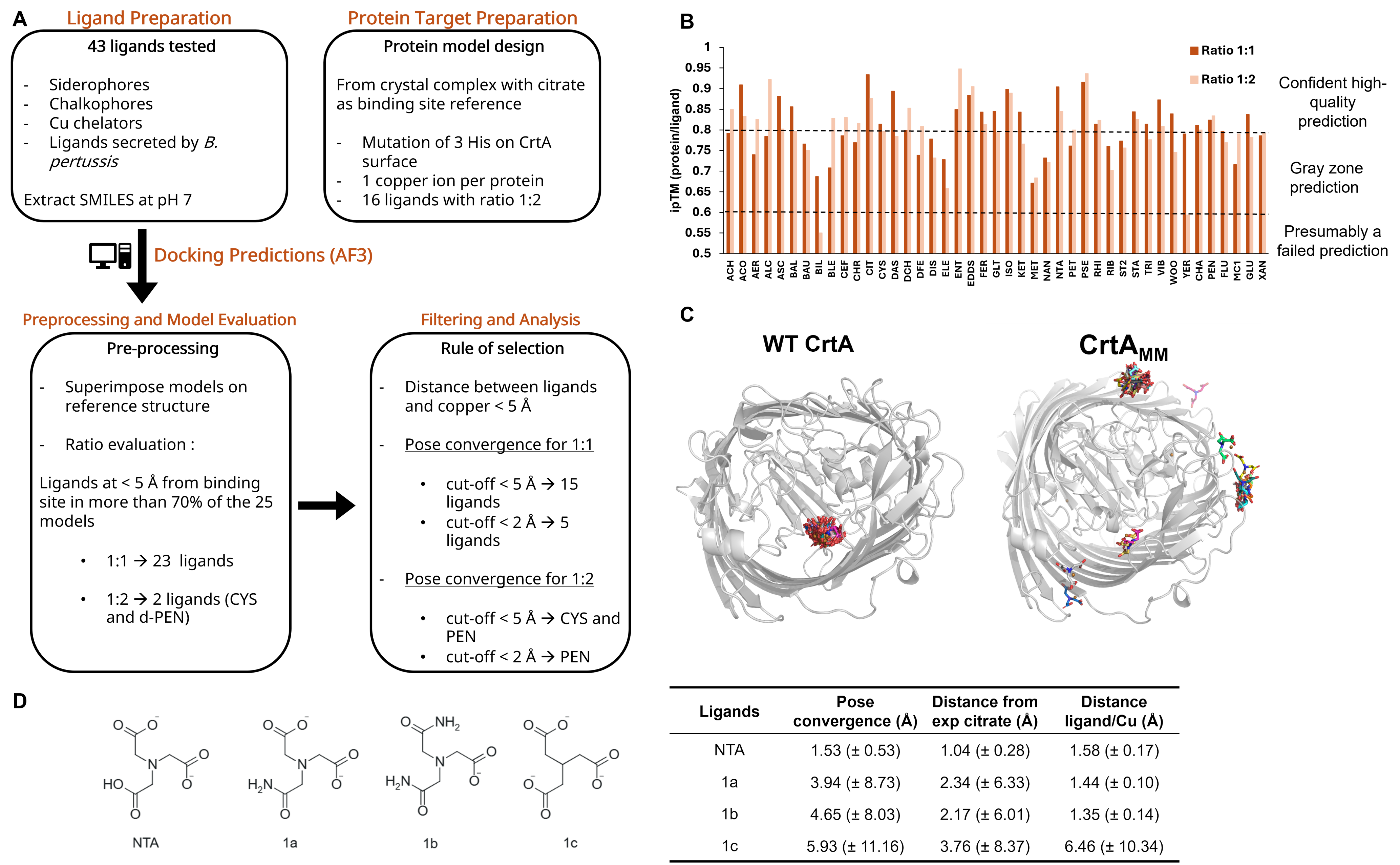


**Figure S10. Analyses of the AF3 models.** **A**, pipeline for the analysis of the models. **B**, ipTM scores obtained for the models of CrtA^Bp^ with various ligands generated by AF3. **C**, effect of the substitutions of the conserved residues with Ala on the model of the CrtA_MM_-NTA-Cu complex. NTA was no longer stabilized in the multiple-mutant protein and adopted a variety of positions around the barrel, with a docking accuracy of 26.975 +/- 16.627Å and a distance from the crystallographic structure of 29.64 +/- 9.57 Å. **D**, *in silico* modifications of NTA and consequences on the pose convergence of the models and on measured distances.

*Supplementary Tables*

**Table S1. List of the TBDTs of known structure (Excel file).** The PDB accession numbers, producing organisms, and co-crystallized ligands are indicated.

**Table S2. Genbank ID numbers of the TBDTs found in labeled clusters.** ID list of all proteins included in clusters labeled in Fig. 1A and in SI Fig. S1A-C.

**Table S3. Genomic analyses of bacterial species harboring *crtA* orthologs (Excel file).** The producing organisms, genetic environments of the *crtA* genes, and the presence of genes of interest in these bacteria are provided. Note that many *crtA* orthologs were found in some non-assembled genomes, hence the possibility that some genes of interest are currently missing.

**Table S4. Analysis of the *crtA* sequences from *B. pertussis* isolates partially or totally sequenced (Excel file).** ‘incomplete’ means that the sequence of the *crtA* gene was not completed in the corresponding isolate, most likely because the genome was not fully sequenced and assembled, and the gene was at the extremity of a contig.

**Table S5. Crystal data, data-collection and refinement statistics.** Values in parentheses are for the highest resolution shell.

| **Data collection** (PDB) | 9RVQ | 9RVX |
| --- | --- | --- |
| Data Set Name | βOG | Citrate |
| Wavelength | 0.91840 | 0.9786 |
| Beamline | ID30B / ESRF | ID30B / ESRF |
| Temperature | 100K | 100K |
| Space Group | *P*2_1_ | *P*1 |
| Unit-cell parameters |  |  |
| a, b, c (Å) | 68.1, 120.1, 119.5 | 67.1, 67.9, 102.5 |
| α, β, γ (°) | 90, 102.24, 90 | 89.3, 80.3, 68.6 |
| Resolution range (Å) | 116 – 2.07 | 100 – 2.29 |
| High resolution range (Å) | 2.31 – 2.07 | 2.59 – 2.29 |
| Observed reflections | 183610 | 66407 |
| No. of unique reflections | 54840 | 38618 |
| Spherical Completeness (%) | 48.3 (8.7) | 52 (8.5) |
| Ellipsoidal Completeness (%) | 88.2 (52.8) | 88.2 (57) |
| CC1/2 | 99.2 (70.5) | 99.3 (73.0) |
| <I/σ(I)> | 5.8 (2) | 4.3 (1.4) |
| *R*_merge_(%) | 11.9 (57.8) | 6.9 (32.8) |
| **Refinement** |  |  |
| Resolution range (Å) | 116 – 2.07 | 26 – 2.29 |
| *R*_workt_ / *R*_free_ | 25.0 / 30.3 | 22.8 / 28.9 |
| No. of non-H atoms: |  |  |
| Protein | 10634 | 10597 |
| βOG | 46 | - |
| Citrate | - | 26 |
| Water | 467 | 104 |
| B-Factors |  |  |
| Protein | 44.5 | 37.6 |
| βOG | 43.4 | - |
| Citrate | - | 28.3 |
| Waters | 32.3 | 24.0 |
| R.m.s. deviation from ideal geometry |  |  |
| Bond lengths (Å) | 0.008 | 0.007 |
| Bond angles (°) | 1.0 | 0.95 |

**Table S6.** **Thermal stability of apo CrtA^Bp^, CrtA^Bp^ + Cu(II), or CrtA^Bp^ + Cu(II) + metabolite**

∆Tm = Tm _CrtA_^Bp^ _+ Cu (II) + Ligand_ - Tm _CrtA_^Bp^ _+ Cu (II)_

**Table S7. List of the molecules tested by AlphaFold3 (Excel file).** The smiles, chemical structure and relevant characteristics are provided for each putative ligand. Note that each ligand was in the protonation state at pH 7 predicted by the Marvin-Sketch software for use by AF3.

**Table S8. Strains, plasmids, synthetic genes and primers used in this work (Excel file).**
